# CircHIPK3 regulates fatty acid metabolism through miR-637/FASN axis to promote esophageal squamous cell carcinoma

**DOI:** 10.1038/s41420-024-01881-z

**Published:** 2024-03-02

**Authors:** Shi-qiang Cao, Song-tao Xue, Wen-juan Li, Guo-sheng Hu, Zhi-gang Wu, Jian-cong Zheng, Shu-liang Zhang, Xiao Lin, Chun Chen, Wen Liu, Bin Zheng

**Affiliations:** 1 Department of Thoracic Surgery, Fujian Medical University Union Hospital, No. 29 Xinquan Road, Fuzhou, Fujian 350001 China; 2 Fujian Key Laboratory of Cardio-Thoracic Surgery, Fujian Medical University, No. 29 Xinquan Road, Fuzhou, Fujian 350001 China; 3 State Key Laboratory of Cellular Stress Biology, School of Pharmaceutical Sciences, Faculty of Medicine and Life Sciences, Xiamen University, Xiang’an South Road, Xiamen, Fujian 361102 China; 4 Fujian Provincial Key Laboratory of Innovative Drug Target Research, School of Pharmaceutical Sciences, Faculty of Medicine and Life Sciences, Xiamen University, Xiang’an South Road, Xiamen, Fujian 361102 China; 5 Xiang An Biomedicine Laboratory, School of Pharmaceutical Sciences, Faculty of Medicine and Life Sciences, Xiamen University, Xiang’an South Road, Xiamen, Fujian 361102 China

**Keywords:** Oesophageal cancer, Cancer epigenetics

## Abstract

The oncogenic role of circRNA in cancers including esophageal cancer (EC) has been well studied. However, whether and how circRNAs are involved in cancer cell metabolic processes remains largely unknown. Here, we reported that circRNA, circHIPK3, is highly expressed in ESCC cell lines and tissues. Knockdown of circHIPK3 significantly restrained cell proliferation, colony formation, migration, and invasion in vitro and inhibited tumor growth in vivo. Mechanistically, circHIPK3 was found to act as a ceRNA by sponging miR-637 to regulate FASN expression and fatty acid metabolism in ESCC cells. Anti-sense oligonucleotide (ASO) targeting circHIPK3 substantially inhibited ESCC both in vitro and in vivo. Therefore, these results uncover a modulatory axis constituting of circHIPK3/miR-637/FASN may be a potential biomarker and therapeutic target for ESCC in the clinic.

## Introduction

Esophageal cancer (EC) is one of the most common malignancies worldwide. It is the sixth leading cause of cancer-related mortality worldwide [1]. It is pathologically divided into esophageal squamous cell carcinoma (ESCC) and esophageal adenocarcinoma (EAC) [2]. The incidence and mortality rates are high in China, and ESCC accounts for 90% of EC [3]. Despite the continuous improvement of diagnosis and treatment, the 5-year survival rate of ESCC is still less than 20% [3, 4]. Unfortunately, the sensitivity and specificity of current screening and treatment methods are not ideal [5, 6]. Therefore, it is urgent to find novel diagnostic markers and drug targets for ESCC diagnosis and treatment, respectively.

Circular RNA (circRNA) was firstly discovered in RNA viruses and the eukaryotic cells [7]. Compared to other linear RNAs, the 5′ and 3′ ends of the circRNA are covalently bound, forming a circular structure without a 5′ end cap and a 3′ poly A tail, which allows the circRNA to escape the degradation by endonuclease and stably exsit in vivo [8–10]. CircRNAs are generally considered to be a by-product of mis-splicing and likely represents errors in splicing [11–14]. With the development of high-throughput sequencing and bioinformatics, a large number of studies have shown that circRNAs are closely related to various diseases [15–20] especially in cancers [21], such as lung cancer [22], breast cancer [23, 24], prostate cancer [25], pancreatic cancer [26], and gastric cancer [27], among others. They are considered as potential biomarkers or therapeutic targets for disease diagnosis and treatment [21, 28]. CircRNAs are known to play an important regulatory role in both genes transcriptional and post-transcriptional regulation [29, 30]. One of the common mechanisms of cytosolic circRNAs function is acting as a ceRNA, competing with mRNA to bind with miRNA and thus regulating downstream gene expression [30, 31]. For example, circ-CDR1as, which harbors more than 70 conserved binding sites for miR-7 and provides a conceptual mechanistic understanding of ceRNA networks [30, 32, 33]. CiRS-7 promotes ESCC growth and metastasis via sponging miR-876-5p to increase MAGEA family expression [34]. CircNTRK2 upregulates NRIP1 expression and promotes ESCC progression via miR-140-3p [35].

CircHIPK3 (circBase ID: hsa_circ_0000284, Position: chr11:33307958-33309057) is generated by back-splicing of exon 2 from HIPK3 [36], which is dysregulated in many types of cancers. Studies have shown that circHIPK3 affects the proliferation, migration, invasion, and other biological behaviors of tumor cells through sponging miRNAs in many types of cancers, such as prostate cancer [37], colorectal cancer [38, 39], gallbladder cancer [40], and cervical cancer [41]. Although studies have shown that circHIPK3 is involved in the development of ESCC, the underlying molecular mechanisms remained to be fully characterized.

Abnormal lipid metabolism is considered to be one of the hallmark pathways in the development of cancer. Fatty acid synthase (FASN) is an intracellular enzyme, promoting the condensation of NADPH-dependent malonyl-coA and acetyl-coA to palmitate, a 16-carbon fatty acid (FA), during the synthesis of endogenous long-chain FAs [42, 43]. In general, only liver, lactating breast, fetal lung, and adipose tissue, but not others, are easy to synthesize fatty acids [44]. Recent studies have shown that FASN is overexpressed in tumors, which may be related to the altered lipid metabolism observed [45–48].

Antisense oligonucleotides (ASOs) are synthetic, short (8–50 base pairs), and single-stranded nucleotide sequences bind to the specific nucleotide sequences of pre-mRNA or mRNA (antisense binding) by means of standard Watson-Crick base pairing, thereby regulating target genes’ function [49–52]. Recently, two antisense nucleotide drugs were approved by the FDA for the treatment of Duchenne muscular dystrophy (DMD) and spinal muscular atrophy (SMA), which is undoubtedly a major breakthrough in the field [49, 53]. Our previous study also indicates that ASO targeting LINC00680 also played an important role in the treatment of ESCC both in vitro and in vivo [54]. Therefore, ASOs provide new ideas for the transformation of basic scientific research into clinical medicine.

## Results

### CircHIPK3 is significantly upregulated in ESCC cell lines and tissues, and knockdown of circHIPK3 suppresses the malignant behaviors of ESCC

**Fig. 1:**
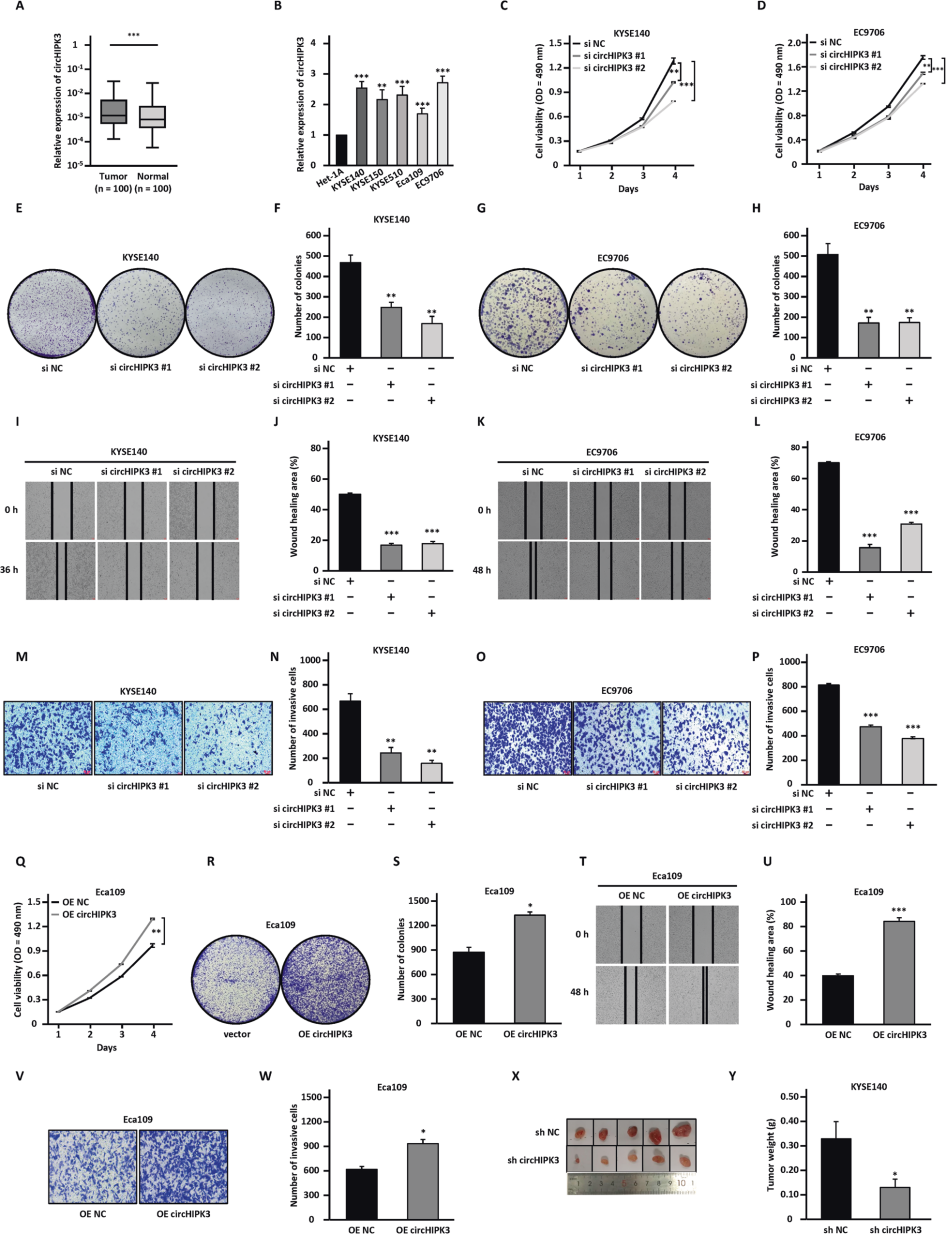
CircHIPK3 is upregulated in ESCC cell lines and tissues promotes cell proliferation, colony formation, migration, and invasion in vitro and tumor growth in vivo. **A** The expression of circHIPK3 was detected by RT-qPCR analysis in 100 pairs of ESCC tumor tissues (Tumor) and matched adjacent normal tissues (Normal). **B** The expression level of circHIPK3 in normal esophageal epithelial cell line and ESCC cell lines as indicated was detected by RT-qPCR analysis. **C**–**P** KYSE140 and EC9706 cells were transfected with negative control siRNA (si NC) or two independent siRNAs targeting circHIPK3 (si circHIPK3 #1 and si circHIPK3 #2) followed by cell proliferation assay (**C**, **D**), colony formation assay (**E**–**H**), wound healing assay (**I**–**L**), and transwell assay (**M**–**P**). **Q**–**W** Eca109 cells were transfected with empty control vector (OE NC) or vector expressing circHIPK3 (OE circHIPK3), followed by cell proliferation assay (**Q**), colony formation assay (**R**, **S**), wound healing assay (**T**, **U**), and transwell assay (**V**, **W**). **X** The sh circHIPK3-transfected cells were injected subcutaneously into nude mice (*n* = 5 per group), and images of xenograft tumors are shown. **Y** The average weight of tumors as shown in (**X**). All experiments were repeated for three times, and representative data is shown (mean ± SD, *P < 0.05, **P < 0.01, ***P < 0.001).

CircHIPK3 (circBase ID: hsa_circ_0000284, Position: chr11:33307958-33309057) is a circRNA that has been reported to be functional important in various types of cancers. In this study, we aimed to investigate the function and molecular mechanism of circHIPK3 in ESCC. We first confirmed circHIPK3 is generated from exon 2 of HIPK3 with 1099 base pair (bp) in length (Fig. S1a, b). CircHIPK3 is much more stable than its parental mRNA, HIPK3 (Fig. S1c, d). Meanwhile, circHIPK3 is resistant to RNase R digestion (Fig. S1e, f). We then investigated its expression in ESCC, finding that it is significantly upregulated in tumor tissues samples compared to adjacent normal tissues (Fig. 1A). Similarly, higher expression of circHIPK3 was observed in multiple ESCC cell lines including KYSE140, KYSE150, KYSE510, ECA109, and EC9706 than normal epithelial cell line, Het-1A (Fig. 1B).

To test whether it is functional important, we designed two independent siRNAs targeting the junction site of circHIPK3 (si circHIPK3 #1 and si circHIPK3 #2). The RT-qPCR analysis results showed that these siRNAs were effective in knocking down circHIPK3 in both KYSE140 and EC9706 cells (Fig. S1g, h). Cell proliferation (Fig. 1C, D) and colony formation (Fig. 1E–H) assay results revealed that the growth of KYSE140 and EC9706 cells was suppressed when circHIPK3 was knocked down. The results from wound healing (Fig. 1I–L) and transwell assay (Fig. 1M–P) indicated that the migration and invasion capabilities of KYSE140 and EC9706 cells were also inhibited when circHIPK3 was knocked down. In contrast, overexpression of circHIPK3 in Eca109 cells (Fig. S1i), which have relatively low levels of circHIPK3 (Fig. 1B), promoted cell growth, migration, and invasion (Fig. 1Q–W). To further evaluate the impact of circHIPK3 on ESCC tumorigenesis in vivo, we first established a KYSE140 cell line stably expressing lentiviral shRNA specifically targeting circHIPK3 (sh circHIPK3) (Fig. S1j). KYSE140 cells with circHIPK3 knockdown grew much slower than control cells (Fig. S1k–m). We then performed xenograft experiment with circHIPK3-knockdown KYSE140 cells and found that the growth of tumors derived from these cells was significantly inhibited (Fig. 1X, Y).

### CircHIPK3 regulates the expression of FASN

**Fig. 2:**
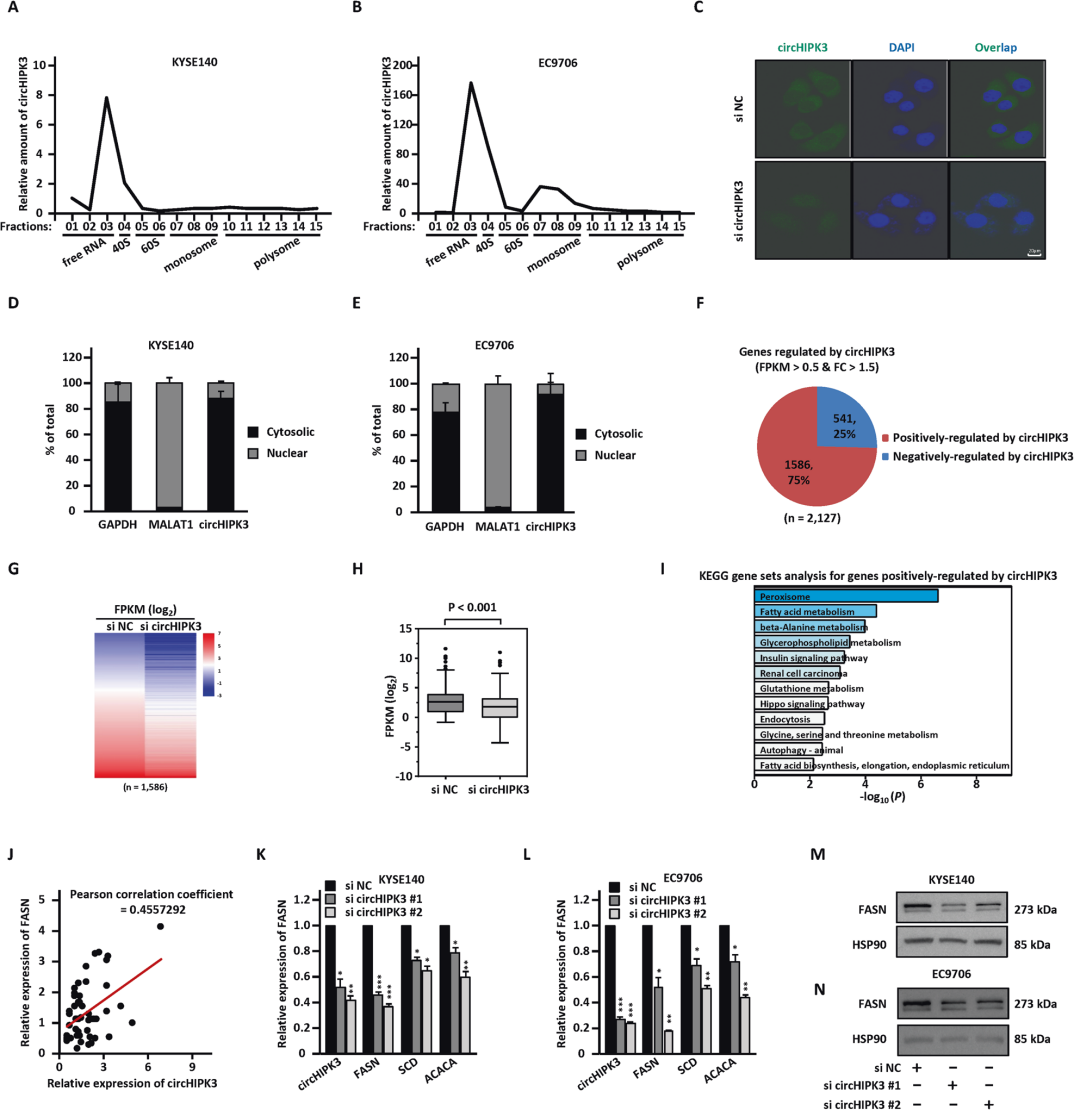
FASN is regulated by circHIPK3. **A**, **B** Polysome profiling was performed to examine the distribution of circHIPK3 in different fractions as indicated in KYSE140 (**A**) and EC9706 (**B**) cells. Fraction 1 to 3: free RNA (ribosome-unbound); Fraction 4: 40S (40S ribosomal subunit); Fractions 5 and 6: 60S (60S ribosomal subunit); Fractions 7 to 9: monosome; Fractions 10 to 15: polysome. **C** RNA-FISH analysis was performed to determine the localization of circHIPK3 in ESCC cells. **D**, **E** KYSE140 (**D**) and EC9706 (**E**) cells were subjected to subcellular fractionation followed by RT-qPCR analysis to determine the subcellular distribution of circHIPK3. **F** KYSE140 cells were transfected with si NC and si circHIPK3 for 72 h followed by RNA-seq analysis, and genes positively and negatively regulated by circHIPK3 are shown by Pie chart. **G, H** The expression of genes positively regulated by circHIPK3 is shown in Heat map (**G**) and box plot (**H**) (unpaired Student’s *t*-test, two-tailed). **I** KEGG gene sets analysis for genes positively regulated by circHIPK3 is shown. **J** The correlation between the expression of FASN and circHIPK3 in ESCC tumor samples (in-house) was analyzed (*n* = 50). **K**–**N** KYSE140 (**K**) and EC9706 (**L**) cells were transfected with si NC and si circHIPK3 for 72 h followed by RT-qPCR (**K**, **L**) and immunoblotting (**M**, **N**) analysis to detect the expression of genes as indicated. All experiments were repeated for three times, and representative data is shown (mean ± SD, *P < 0.05, **P < 0.01, ***P < 0.001).

To understand the molecular mechanisms by which circHIPK3 promotes the malignant behaviors of ESCC, we first confirmed that circHIPK3 is a real non-coding RNA through polysome profiling experiments (Fig. 2A, B). Then, RNA-FISH (Fig. 2C) and cellular fractionation assay (Fig. 2D, E) results demonstrated that circHIPK3 is mainly located in the cytoplasm of cells.

In order to explore the downstream target genes of circHIPK3 in regulating the malignant behaviors of ESCC, transcriptomic analysis was performed using KYSE140 cells with or without circHIPK3 knockdown. The results showed that a total of 1586 genes were positively and 541 genes were negatively regulated by circHIPK3 (Fig. 2F–H). For a large number of positively regulated target genes, KEGG pathway analysis results revealed that gene sets, such as peroxisome, fatty acid metabolism, beta-alanine metabolism, glycerophospholipid metabolism, and insulin signaling pathway, were the most enriched (Fig. 2I). Cytosolic circRNA may regulate target genes through the ceRNA mechanism. We therefore explored the possibility that circHIPK3 could regulate the expression of target genes by sponging miRNAs. Three different algorithms, TarPmiR [55], miRanda [56], and RNAhybrid [57], were used to predict the potential miRNAs that could bind circHIPK3, and then the predicted miRNAs with high confidence were overlapped. The circRNA-miRNA-mRNA network was constructed to connect circHIPK3 with its target gene through miRNAs (Fig. S2a). There were altogether 9 miRNAs, including miR-637, miR-134-5p, miR-3132, miR-193a-3p, miR-193b-3p, miR-3529-5p, miR-5087, miR-6788-3p, and miR-3945, as well as 54 target genes involved in the ceRNA network. Target genes directly linked to fatty acid metabolism, such as FASN, SCD, and ACACA, caught our attention as they were also enriched in KEGG pathway analysis. These genes were significantly upregulated in ESCC (Table S2). Furthermore, we examined the correlation between the expression of these target genes and circHIPK3 in ESCC tissues, finding that FASN and circHIPK3 expression were the most correlated compared to SCD and ACACA (Fig. 2J and Fig. S2b, c). RT-qPCR analysis resulted showed that FASN was the most regulated compared to SCD and ACACA in response to circHIPK3 knockdown in both KYSE140 cells and EC9706 cells (Fig. 2K, L). Furthermore, immunoblotting analysis results confirmed that the expression of FASN was significantly decreased after circHIPK3 knockdown, suggesting that FASN may be the downstream target gene of circHIPK3 (Fig. 2M, N).

### FASN promotes the malignant behaviors of ESCC cells

**Fig. 3:**
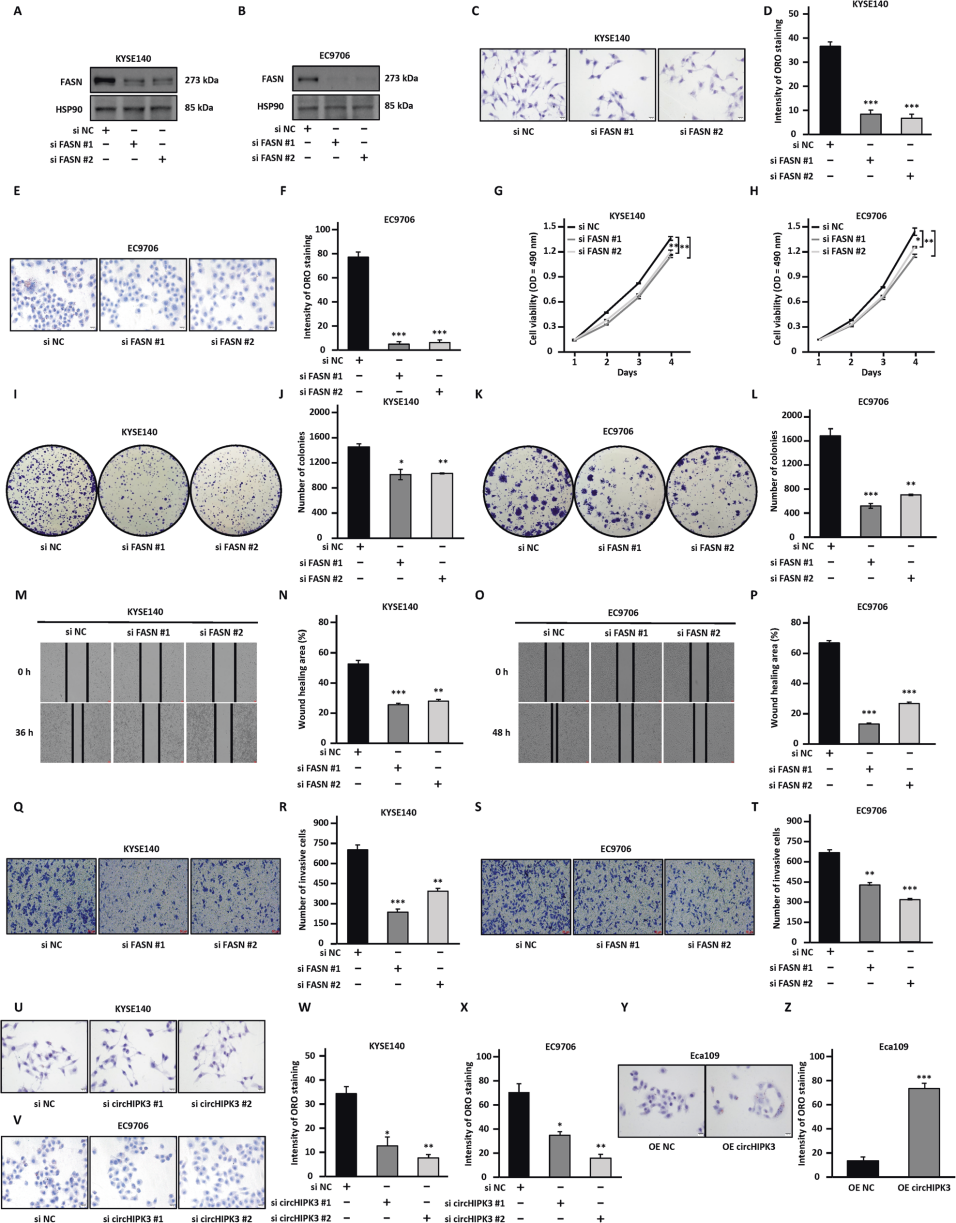
FASN promotes the malignant behaviors of ESCC cells. **A**–**T** KYSE140 and EC9706 cells were transfected with negative control siRNA (si NC) or two independent siRNAs targeting FASN (si FASN #1 and si FASN #2) followed by immunoblotting analysis (**A**, **B**), Oil Red O staining (**C**–**F**), cell proliferation assay (**G, H**), colony formation assay (**I**–**L**), wound healing assay (**M**–**P**), and transwell assay (**Q**–**T**). **U**–**X** KYSE140 and EC9706 cells were transfected with negative control siRNA (si NC) or two independent siRNAs targeting circHIPK3 (si circHIPK3 #1 and si circHIPK3 #2) followed by Oil Red O staining. **Y**, **Z** Eca109 cells were transfected with empty vector control or vector expressing circHIPK3 followed by Oil Red O staining. All experiments were repeated for three times, and representative data is shown (mean ± SD, *P < 0.05, **P < 0.01, ***P < 0.001).

To test whether FASN is a functional target of circHIPK3, we first explored whether FASN is involved in the malignant behaviors of ESCC cells. KYSE140 and EC9706 cells were transfected with control siRNA (si NC) or two independent FASN targeting siRNA (si FASN #1 and si FASN #2), and the knockdown efficiency was verified by RT-qPCR (Fig. S3a, b) and western blotting (Fig. 3A, B) analysis. As expected, knocking down of the FASN resulted in a decrease in the signals from Oil Red O staining (Fig. 3C–F). Cell proliferation (Fig. 3G, H), colony formation (Fig. 3I–L), wound healing (Fig. 3M–P), and transwell (Fig. 3Q–T) assay results showed that knocking down FASN resulted in significant inhibition of cell proliferation, colony formation, migration, and invasion ability of KYSE140 and EC9706 cells. The above experiments suggested that FASN has similar functions as circHIPK3. Accordingly, Oil Red O staining signals were decreased after circHIPK3 knockdown, while increased upon circHIPK3 overexpression in Eca109 cells (Fig. 3U–Z).

### FASN is a key downstream target of circHIPK3 to promote ESCC progression

**Fig. 4:**
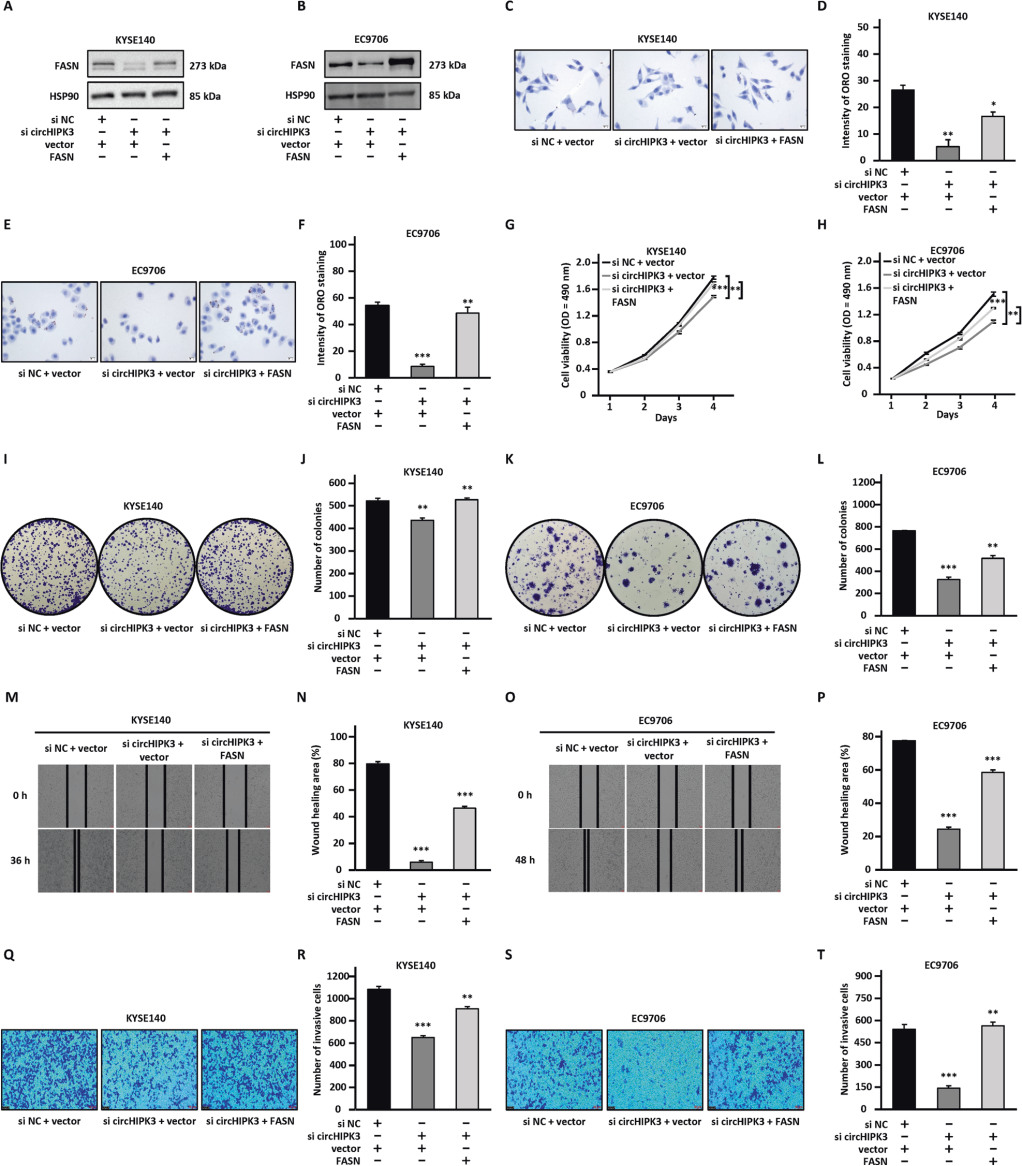
FASN is a key downstream target gene of circHIPK3. **A**–**T** KYSE140 and EC9706 cells were transfected with negative control siRNA (si NC) or siRNA targeting circHIPK3 (si circHIPK3) in the presence or absence of FASN expression vector, followed by immunoblotting analysis (**A**, **B**), Oil Red O staining (**C**–**F**), cell proliferation assay (**G**, **H**), colony formation assay (**I**–**L**), wound healing assay (**M**–**P**), and transwell assay (**Q**–**T**). All experiments were repeated for three times, and representative data is shown (mean ± SD, *P < 0.05, **P < 0.01, ***P < 0.001).

In order to further test whether FASN is key downstream target of circHIPK3, we carried out rescue experiments, in which KYSE140 and EC9706 cells were transfected with FASN overexpression vector while knocking down circHIPK3 (Fig. 4A, B, and Fig. S4a, b). The effects of circHIPK3 silencing on Oil Red O staining signals as well as the inhibition of cell proliferation, colony formation, migration, and invasion ability were attenuated by FASN overexpression (Fig. 4C–T).

### CircHIPK3 regulates the expression of FASN via sponging miR-637

**Fig. 5:**
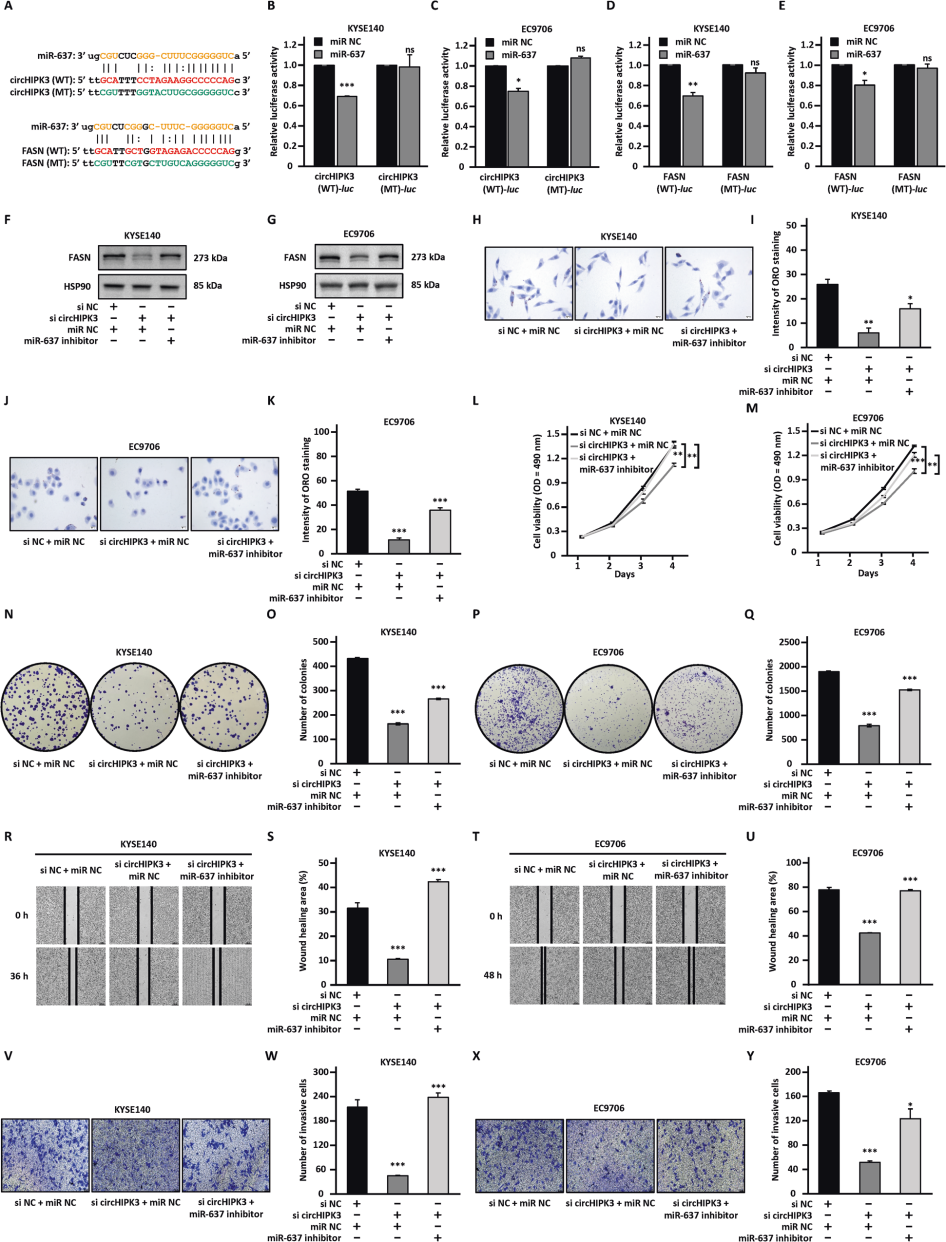
CircHIPK3 serves as a ceRNA to sponge miR-637 to regulate the expression of FASN and the malignant behavior of ESCC cells. **A** Schematic illustration of circHIPK3 and FASN wild-type (WT) and mutant (MT) with the miR-637 binding site mutated. **B**, **C** KYSE140 and EC9706 cells were co-transfected with luciferase reporter vectors containing wild-type (WT-*luc*) or mutated (MT-*luc*) circHIPK3 in the presence of negative control miRNA mimic (miR-NC) or miR-637 mimic followed by dual-luciferase reporter assay. **D**, **E** KYSE140, and EC9706 cells were co-transfected with luciferase reporter vectors containing wild-type (WT-*luc*) or mutated (MT-*luc*) FASN in the presence of miR-NC or and miR-637 mimic followed by dual-luciferase reporter assay. **F**–**Y** KYSE140 and EC9706 cells were transfected with negative control siRNA (si NC) or siRNA targeting circHIPK3 (si circHIPK3) in the presence or absence of miR-637 inhibitor, followed by immunoblotting analysis (**F**, **G**), Oil red O staining (**H**–**K**), cell proliferation assay (**L**, **M**), colony formation assay (**N**–**Q**), wound healing assay (**R**–**U**), and transwell assay (**V**–**Y**). All experiments were repeated for three times, and representative data is shown (mean ± SD, *P < 0.05, **P < 0.01, ***P < 0.001).

The ceRNA network predicted that circHIPK3 could regulate the expression of FASN by sponging miR-637. MiR-637 has been reported to be involved in many human cancers [58]. And it was predicted to bind to the circHIPK3 sequence and the 3′UTR of FASN through starBase (Fig. 5A). The wild-type linear sequence of circHIPK3 or 3′UTR of FASN (WT-*luc*) as well as the corresponding mutant form with the predicted miR-637 binding site mutated (MT-*luc*) were cloned into luciferase reporter vectors and transfected into KYSE140 and EC9706 cells together with control miRNA (miR-NC) or miR-637 mimic. The results showed that overexpression of miR-637 resulted in significantly reduced luciferase activity of the plasmids carrying circHIPK3 (WT)-*luc* and FASN (WT)-*luc* compared with the miR-NC group, while there was no significant change in luciferase activity of the plasmids carrying circHIPK3 (MT)-*luc* or FASN (MT)-*luc* (Fig. 5B–E). To support that circHIPK3 could serve as a sponge for miR-637, the copy number of circHIPK3 and miR-637 was comparable, which was approximately 91 and 40 copies per cell, respectively, in KYSE140 cells (Fig. S5a–c). Meanwhile, 215 copies of circHIPK3 and 59 copies of miR-637 per cell were detected in EC9706 cells (Fig. S5d).

To test whether circHIPK3 regulation of FASN and the malignant behaviors of ESCC is through miR-637, we transfected si NC or si circHIPK3 into KYSE140 and EC9706 cells with or without miR-637 inhibitors. When circHIPK3 was knocked down, FASN expression was inhibited at both mRNA and protein levels, which was significantly counteracted by miR-637 inhibitor (Fig. 5F, G and Fig. S5e, f). In addition, miR-637 inhibitor largely diminished the effects of circHIPK3 silencing on Oil Red O staining signals as well as proliferation, colony formation, migration, and invasion ability of KYSE140 and EC9706 cells (Fig. 5H–Y).

### CircHIPK3 is a potential therapeutic target for ESCC

**Fig. 6:**
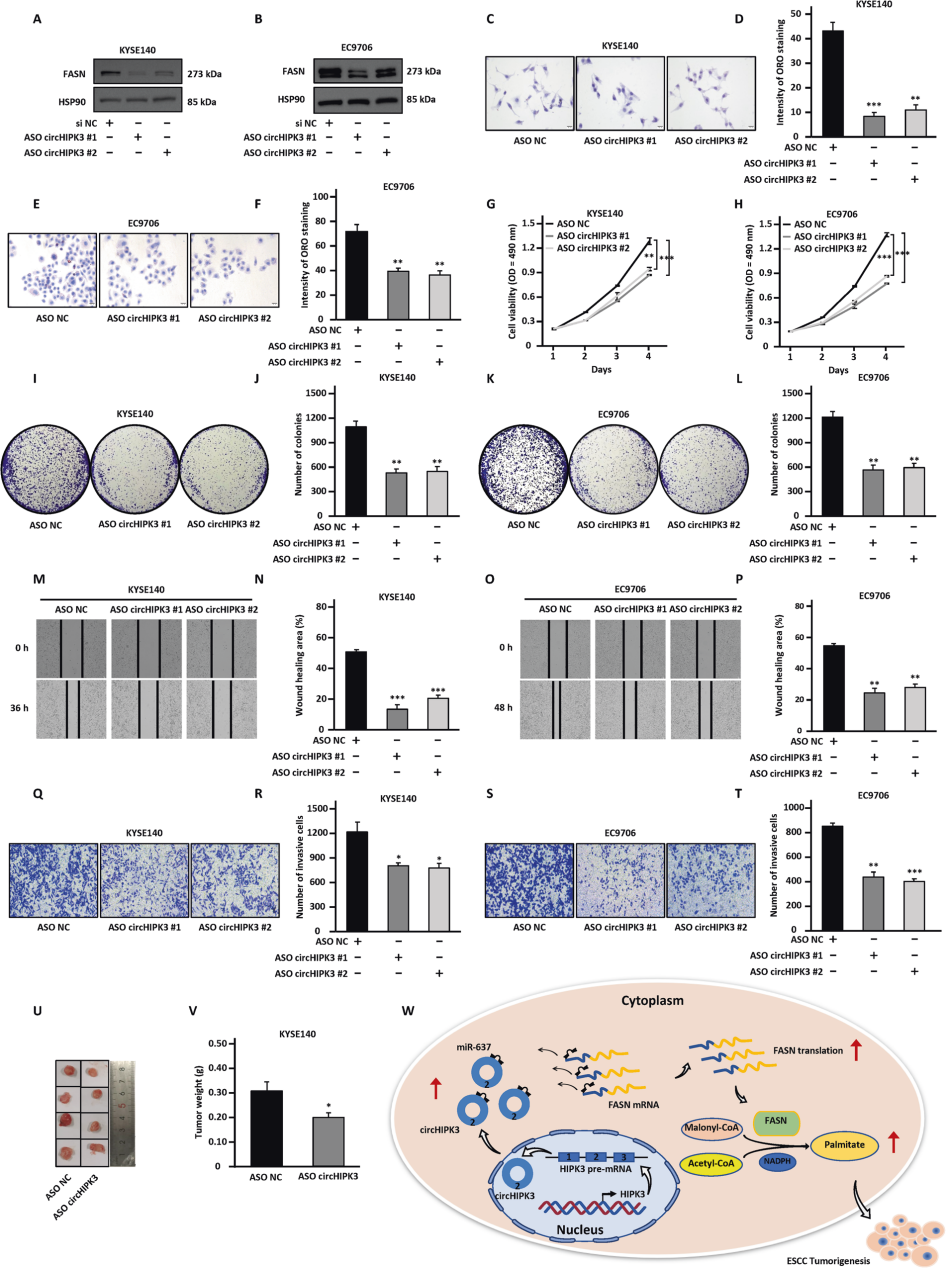
CircHIPK3 is a potential therapeutic target in ESCC. **A**–**T** KYSE140 and EC9706 cells were transfected with negative control ASO (ASO NC) or two independent ASOs specifically targeting circHIPK3 (ASO circHIPK3 #1 and ASO circHIPK3 #2), followed by immunoblotting analysis (**A**, **B**), Oil Red O staining (**C**–**F**), proliferation assay (**G**, **H**), colony formation assay (**I**–**L**), wound healing assay (**M**–**P**), and transwell assay (**Q**–**T**). **U** KYSE140 cells infected with sh NC or sh circHIPK3 were subcutaneously injected into nude mice. When tumor size reached about 100 mm^3^, ASO NC or ASO circHIPK3 were delivered by intratumor injection. Mice were euthanized three weeks later and tumors were removed for imaging. **V** The weight of tumor in (**U**) is shown. **W** The proposed model for circHIPK3 function in ESCC. The highly expressed circHIPK3 acts as a sponge for miR-637 to relieve the inhibition of miR-637 on FASN expression, promoting ESCC tumorigenesis. All experiments were repeated for three times, and representative data is shown (mean ± SD, *P < 0.05, **P < 0.01, ***P < 0.001).

Currently, antisense oligonucleotides (ASOs) have received increasing attention due to their ability to specifically target and degrade specific RNAs, which has been demonstrated in vitro and in vivo [54, 59, 60]. The upregulation of circHIPK3 in ESCC tumor tissues and its significant effects on the malignant phenotypes of ESCC prompted us to design two independent ASOs targeting circHIPK3 (ASO circHIPK3 #1 and ASO circHIPK3 #2) and test its effects on the malignant behaviors of ESCC. Knockdown efficiency of ASO circHIPK3 was detected by RT-qPCR analysis (Fig. S6a, b). Moreover, the changes of FASN protein level after circHIPK3 knockdown were detected by western blotting (Fig. 6A, B). ASO circHIPK3 transfection led to the decrease of Oil Red O staining compared to negative control ASO (ASO NC) (Fig. 6C–F). Results from cell proliferation (Fig. 6G, H), colony formation (Fig. 6I–L), wound healing (Fig. 6M–P), and transwell (Fig. 6Q–T) assays showed that, compared with the negative control group, the ability of cell proliferation, colony formation, migration, and invasion were significantly weakened after ASO interference. To evaluate tumor growth in vivo, mice were inoculated with KYSE140 cells and randomly divided into two groups, which were then treated with ASO NC or ASO circHIPK3 every 5 days. Tumor growth was significantly inhibited in the ASO circHIPK3-treated group compared with the ASO NC group (Fig. 6U, V). In summary, the current data suggest that circHIPK3 may be a therapeutic target for ESCC, and ASO targeting circHIPK3 provides a promising approach for the treatment of ESCC patients.

## Discussion

Recently, the role of circRNA in the occurrence and development of cancer has attracted extensive attention. The action mechanisms of circRNAs in cancer development are versatile, such as ceRNA, protein scaffold, gene transcriptional regulation, and translation regulation. The role of circHIPK3 in cancers, such as gastric cancer, breast cancer, colon cancer, lung cancer, renal cancer, osteosarcoma, melanoma, prostate cancer, thyroid, bladder cancer, pancreatic cancer, and ESCC, has been well documented [61, 62], which suggest that has important roles in tumors. However, its role in the regulation of fatty acid metabolism in ESCC has not been reported. We confirmed that the expression level of circHIPK3 was significantly upregulated in ESCC cell lines and tissue samples compared with normal esophageal cell line and adjacent paracancerous tissues. We then carried out a series of experiments in vitro and in vivo to further determine that circHIPK3 can promote the malignant behaviors of ESCC cells. Through transcriptomic analysis, we found that, among the enriched molecular pathways for genes regulated by circHIPK3, the fatty acid metabolism pathway was of particular interest. Previous studies have shown that dysregulation of fatty acid metabolism is one of the hallmarks in a variety of cancers including ESCC [63, 64]. We confirmed that the expression level of circHIPK3-target gene FASN, one of the key enzymes involved in fatty acid metabolism, is highly expressed in ESCC tissue samples, and knocking down FASN had similar effects on the malignant behaviors of ESCC. FASN is a well-studied fatty acid synthesis-related enzyme that catalyzes acetyl-CoA and malonyl-CoA to generate endogenous fatty acids. Under normal circumstances, except for liver, adipose tissue, lactating mammary gland, and fetal lung, the activity of FASN in other tissue cells is very low. They mainly use exogenous fatty acids to synthesize biofilms and inflammatory mediators, and hardly need to synthesize endogenous fatty acid. The fatty acids of tumor cells mainly come from endogenous fatty acid synthesis [65, 66]. In recent years, a large number of studies have found that FASN is overexpressed in solid tissue tumors such as esophageal cancer, breast cancer, prostate cancer, ovarian cancer, endometrial cancer, gastric cancer, malignant melanoma, and is associated with the malignant phenotype and poor clinical characteristics of tumors [43, 67–70]. As a key enzyme in fatty acid synthesis, FASN may serve as a new target for antitumor therapy and play an important role in tumor diagnosis and treatment [71]. The antitumor properties induced by circHIPK3 silencing could be greatly attenuated by FASN overexpression. From the above results, we concluded that circHIPK3 promotes ESCC progression, at least partially, by stimulating FASN overexpression. It should be noted that the expression of SCD and ACACA, though to a lesser extent, was also decreased in response to circHIPK3 knockdown. Therefore, whether SCD and ACACA can also serve as downstream target genes of circHIPK3 in regulating fatty acid metabolism to promote ESCC is worthy of future investigation. Predictions from ceRNA networks combined with bioinformatic methods and luciferase reporter assays indicated that miR-637 could bind to circHIPK3 and FASN. So far, miR-637 has been shown to be involved in a variety of malignant behaviors [58, 72, 73]. For example, in triple-negative breast cancer, ectopic expression of miR-637 significantly attenuated the promotion of proliferation, migration, and invasion induced by upregulation of circSEPT9 in TNBC cells [73]. Our study also found that miR-637 knockdown using miR-637 inhibitor could reverse the decrease in FASN expression induced by circHIPK3 knockdown. Furthermore, the antitumor effect mediated by circHIPK3 knockdown was significantly reversed after introduction of miR-637 inhibitor in ESCC cells. Taken together, these results suggest that circHIPK3 may accelerate ESCC progression by acting as a sponge for miR-637 to relieve its repressive effect on FASN expression.

Current cancer treatment strategies using antibodies or small molecules have limitations, such as lack of antibody selectivity or specificity, low target expression in cells, poor drug bioavailability, and severe toxic side effects. In contrast, ASOs specifically target the underlying pathogenic causes of cancer from the RNA level, thereby regulating the expression of key pathogenic proteins that are dysregulated in cancer progression, providing a better approach for the field of molecular therapy. Therefore, the potential of circHIPK3 as a circRNA-based cancer therapeutic target deserves further exploration. Here, we found that ASO targeting circHIPK3 significantly affected fatty acid metabolism in ESCC cells and inhibited cell proliferation, colony formation, migration, and invasion abilities in vitro and tumor growth in mouse xenograft models. The above results suggest that ASO circHIPK3 may be a promising therapeutic approach to delay circHIPK3-promoted ESCC progression. Our results shed new light on the dysregulated circHIPK3/miR-637/FASN axis during ESCC development and foresee that circHIPK3 may serve as a potential diagnostic marker and an effective therapeutic target for ESCC (Fig. 6W). However, there are several questions remain to be fully addressed in the current manuscript. Firstly, we only explored the molecular mechanism of circHIPK3 acting as a miRNA sponge to promote ESCC, while whether it can function through other mechanisms such as protein scaffold remain unknown. Secondly, how FASN reprograms fatty acid metabolism in ESCC cells is underexplored. Thirdly, whether circHIPK3 can serve as a prognosis marker for patients with ESCC remains unclear due to that tracking the survival information for some of the patients are still ongoing.

In conclusion, our experiments show that circHIPK3 is highly expressed in ESCC and promotes ESCC through a circHIPK3/miR-637/FASN axis. These findings reveal that circHIPK3 may become a potential diagnostic biomarker and therapeutic target for ESCC.

## Materials and methods

### Clinical tissue specimens

A total of 100 pairs of ESCC tumor tissues and adjacent normal epithelial tissues were obtained from ESCC patients who underwent resection surgery at thoracic surgery department of Fujian Medical University Union Hospital between 2015 and 2018. All clinicopathological diagnosis were further confirmed by two veteran pathologists according to the American Joint Committee on Cancer (AJCC) and International Union Against Cancer (UICC) tumor staging system. After surgical resection, all specimens were placed in liquid nitrogen immediately and then stored at −80 °C until RNA extraction., histologic.

### Cell culture

The human ESCC cell lines (KYSE140, KYSE510, KYSE150, ECa109, and EC9706) and human normal esophageal epithelial cell line (HET-1a) were preserved by our laboratory for years free of mycoplasma contamination, and were cultured in RPMI1640 medium (Biological Industries) or Dulbecco’s modified Eagle’s medium (DMEM, Biological Industries) supplemented with 10% fetal bovine serum (FBS, Biological Industries) and 1% penicillin/streptomycin mixture (Biological Industries) as supplements. All cell lines were cultured in a humidified incubator containing 5% CO_2_ at 37 °C.

### RNA isolation, reverse transcription (RT), and real-time quantitative polymerase chain reaction (RT-qPCR)

Total RNA from each specimen was extracted by TRIzol reagent (Takara) according to the manufacturer’s instructions. For mRNA and circRNA, reverse transcription was performed using GoScript™ Reverse Transcription Mix (Promega) with random primers. The RT-qPCR assay was carried out with Hieff® qPCR SYBR Green Master Mix (Yeasen) and AriaMx Real-Time PCR machine (Agilent Technologies). All reactions were repeated in three independent experiments with β-actin as an internal control. The expression levels of the genes tested were normalized to the endogenous control, and the relative quantification method (2^-ΔΔCt^) was used for the calculation of fold-change values. Primers used for amplifying specific genes in this study are shown in Supplementary Table 1 (Table S1).

### RNase R treatment

Total RNA was incubated at 37 °C for 2 h with 2 U/μg RNase R (Epicentre Biotechnologies, Madison, WI, USA), and equal amount of total RNA after treatment was subjected to RT-qPCR analysis.

### Actinomycin D treatment

KYSE140 and EC9706 cells were seeded in six-well plates and treated with 100 ng/mL actinomycin D (Amresco, Solon, OH, USA) to inhibit new RNA synthesis for 0 to 10 h. Then, circHIPK3 and HIPK3 mRNA were detected by RT-qPCR analysis.

### Cell transfection, lentivirus packaging, and infection

The specific siRNAs, miRNA inhibitors, miRNA mimics, and ASOs as well as the matched non-specific control were designed and synthesized by RiboBio (Guangzhou, China). Cells were seeded (3 × 10^5^ cells per well) onto the 6-well culture plate and were transfected with specific siRNAs (or miRNA inhibitors, miRNA mimics, ASOs) or the non-specific control by Lipofectamine 2000™ transfection reagent (Invitrogen) according to the manufacturer’s instructions. The transfection efficiency was verified by RT-qPCR. The shRNA targeting circHIPK3 were designed and cloned into the pLKO.1 vector (GenePharma, Shanghai, China). Lentiviruses were packaged in 293 T cells according to the manufacturer’s instructions and cells were selected using puromycin for one week. Linear sequence of circHIPK3 was cloned into the lentiviral expression vector pLO5-ciR (Gysey Biotechnology, China). The targeting sequencing of siRNAs, shRNAs, miRNA inhibitors, miRNA mimics, and ASOs were included in Supplementary Table 1 (Table S1).

### Cell proliferation assay

Cells were plated in triplicate at 2 × 10^3^ cells/well in 96-well plates in culture media and incubated at 37 °C with 5% CO_2_. After incubation for 0, 24, 48, 72, or 96 h, 20 μL of MTS reagent from the CellTiter96^®^Aqueous One Solution Cell Proliferation Assay kit (Promega) was added into each well, following incubation at 37 °C with 5% CO_2_ for 1 h. The absorbance at 490 nm was determined using Multiskan MK3 Microplate Reader (Thermo Fisher, Waltham, MA, USA).

### Colony formation assay

The transfected cells were re-seeded in six-well plates (about 2000 cells per well) and incubated at 37 °C for 10–14 days. After washing with PBS and fixing in methanol for 20 min at room temperature, the cells were stained with 0.1% crystal violet solution for 20 min at room temperature, and the colonies were then counted.

### Wound healing assay

The transfected cells were re-seeded in six-well plates and incubated until the cells were about 90% confluent. Then, scratch wounds were produced in each well using a 10 μL plastic pipette tip. After scratching (recorded as 0 h), the scratch was monitored and photographed at 0, 12, 24, 36, or 48 h. Cell migration was quantified by measuring the distance between the advancing margins of cells in three randomly selected microscopic fields at each time point.

### Transwell assay

Transwell invasion chambers with 50 ng/mL Matrigel (Corning) were prepared according to manufacturer’s instructions. About 1 × 10^5^ cells suspended in 250 μL serum-free medium were placed in the upper chambers and 500 μL medium with 10% FBS was added to the lower chambers. After culturing for 24–36 h, the invasive cells in the lower chambers were fixed with methanol for 20 min at room temperature and then stained with 0.1% crystal violet solution for another 20 min at room temperature. The cells remaining in the upper chamber were removed by cotton swabs. For quantification, cell images were captured in five randomly chosen fields under a microscope and counted.

### Xenograft assay

Male nude mice (4–5 weeks) were maintained in laminar flow cabinets under specific pathogen-free conditions in accordance with a protocol approved by the Animal Ethics Committee of Xiamen University. KYSE140 cells (4 × 10^6^ cells in 0.1 ml PBS) transfected with sh NC, sh circHIPK3 were subcutaneously injected into the armpit of nude mice (10 nude mice were randomly divided into 2 groups). For ASOs treatment, ASOs were delivered by intratumor injection every 5 days at 5 nmol/mouse (100 μL, 50 mM ASO or ASO NC in PBS) with a tumor size about 100 mm^3^. Three weeks later, the mice were euthanized using pentobarbital and the tumors were removed for analysis. Standard of blinding and randomization was complied with in this study.

### Subcellular fractionation

The ESCC cells were harvested and lysed successively, and the cytoplasm fraction and the nucleus fraction were collected respectively. Briefly, the ESCC cells were washed three times with cold PBS, collected, spun down, re-suspended with cold buffer I (10 mM Hepes, pH 8.0, 10 mM KCl, 1.5 mM MgCl_2_, 1 mM DTT) supplemented with a cocktail of protease inhibitors, and incubated on ice for 15 min. Igepal-CA630 was added at a final concentration of 1% and vortexed for 10 seconds. Nuclei were collected by centrifugation (~21,100 × *g*) for 2~3 min. The obtained supernatant was cytosolic fraction. The nuclei were then dissolved in cold buffer II (20 mM Hepes, pH 8.0, 25% (v/v) glycerol, 1.5 mM MgCl_2_, 420 mM NaCl, 1 mM DTT, 0.2 mM EDTA) supplemented with protease inhibitors, followed by vigorous rotation at 4 °C for 30 min and centrifuged at maximum speed for 15 min. The resultant supernatant was nuclear fraction. Both cytosolic and nuclear RNAs were extracted using TRIzol reagents (Takara). The relative levels of circHIPK3 in cytoplasm and nucleus were calculated by RT-qPCR using CT values method. GAPDH and MALAT1 were served as the internal control for the cytoplasmic and nuclear RNA, respectively. Primers are listed in Supplementary Table 1 (Table S1).

### RNA-Fluorescence in situ hybridization (RNA-FISH)

Cy5-labeled RNA oligo probes against the splice junction of circHIPK3 were designed and synthesized by Sangon Biotech (Shanghai, China). The hybridization reactions were performed using a Fluorescent in situ Hybridization Kit (Sangon Biotech) according to the manufacturer’s protocol.

### MiRNA targets prediction of circHIPK3

To construct a competing endogenous RNA (ceRNA) network, three independent algorithms, TarPmiR (probability of target site, -p 0.8) [55], miRanda (sequence align score, -sc 150) [56], and RNAhybrid (minimal free energy, -e −23) [57], were used to predict the miRNAs that could bind to circHIPK3 based on miRbase. Then, the miRNAs (miR-637, miR-3529-5p, miR-3945, miR-5087, miR-134-5p, miR-3132, miR-193b-3p, miR-6788-3p, miR-193a-3p) were predicted for follow-up analysis. And the mRNA targets that were positively regulated by circHIPK3 were selected to construct the ceRNA network by Cytoscape [74].

### Dual-luciferase reporter assay

The circHIPK3 and the 3’ UTR regions of FASN with the potential miR-637 binding sites as well as its mutant forms were designed, synthesized and inserted into psi-check2 vectors (Sangon Biotech), which were termed as circHIPK3 (WT)-*luc*, FASN (WT)-*luc*, circHIPK3 (MT)-*luc*, and FASN (MT)-*luc*. The relative luciferase activity was performed with the Dual Luciferase Assay Kit (Promega, USA) according to the manufacturer’s instructions.

### Western blotting

The collected cells were lysed with RIPA extraction reagent (Beyotime) with PMSF protease inhibitor (Beyotime), and the protein concentration was determined using BCA Protein Detection Kit (Beyotime). The proteins were separated by SDS-PAGE, and transferred onto the polyvinylidene fluoride (PVDF) membrane (Millipore). The membranes were blocked using QuickBlock™ (Beyotime) according to the manufacturer’s instructions and incubated with primary antibody overnight at 4 °C followed by secondary antibodies for 1 h at room temperature. Immunoreactive bands were detected using a chemiluminescent ECL reagent (Millipore). HSP90 was used as an internal control. Antibodies against the following proteins were used: FASN (A21182, ABclonal), HSP90 (D120009, Sangon Biotech). Anti-rabbit IgG (#7074, Cell Signaling Technology) was used as secondary antibody.

### Oil Red O (ORO) staining

Cells were seeded in a cell culture dish with glass slide at a density of approximately 1 × 10^5^ cells per well and incubated overnight at 37 °C with 5% CO_2_. Intracellular fatty acid was determined with the Oil Red O Stain Kit (Solarbio, Beijing) according to the manufacturer’s instructions. The cells were fixed by ORO fixative for 10–15 min, and the glass slide were taken out from the culture dish and air-dried for 10–15 min. The glass slides were then immersed in freshly prepared ORO staining solution for 15 min, rinsed with 60% isopropanol for 20–30 s once and water once, followed by a quick wash in distilled water. Subsequently, the nuclei were stained with Mayer’s hematoxylin for 2 min. Finally, the slides were immersed in ORO buffer solution for 1 min. Images were then taken with an inverted microscope (Leica).

### Copy number detection

The exact copy numbers of circHIPK3 and miR-637 per KYSE140 and EC9706 cell were quantified by RT-qPCR assay. In this assay, the serially diluted RT-PCR products were used as templates to formulate standard curves, and then, the exact copies of circHIPK3 and miR-637 per cell were calculated using the online tool (https://cels.uri.edu/gsc/cndna.html).

### Statistical analysis

All data are shown as the mean ± the standard deviation (SD) from at least three independent experimental replicates. Two-tailed Student’s *t*-test was used for comparisons between two groups, and one-way ANOVA was used for multiple group comparisons. Pearson or Spearman correlation analysis was used to analyze the correlation between the two groups. Differences were considered significant if *P < 0.05, **P < 0.01, and ***P < 0.001.

### Supplementary information

Supplementary figure legends

Supplementary table legends

Table S1

Table S2

Fig. S1

Fig. S2

Fig. S3

Fig. S4

Fig. S5

Fig. S6

Original Data File

## Data Availability

All the data supporting the findings of this study are available within the article and its additional files and from the corresponding author upon reasonable request.
