# Supplementary material for: CircHIPK3 regulates fatty acid metabolism through miR-637/FASN axis to promote esophageal squamous cell carcinoma
Source: Cell Death Discov. 2024 Mar 2;10:110. doi: 10.1038/s41420-024-01881-z (PMC10908791; doi:10.1038/s41420-024-01881-z)
Supplement: Supplementary file 1 — Supplementary figure legends [file 41420_2024_1881_MOESM1_ESM.docx]

**Supplementary figure legends**

**Figure S1. The general characteristics of circHIPK3**

**(a)** The linear or circular HIPK3 were amplified by PCR using primer sets as indicated and cDNA from KYSE 140 and EC 9706 cells as template. gDNA was used as a control. DNA marker is shown on the left. Base pair, bp. **(b)** PCR products from circHIPK3 amplification was subjected to sanger sequencing, and sequencing results for the junction region are shown at the bottom. **(c-d)** The expression of linear and circular HIPK3 in KYSE140 **(c)** and EC9706 **(d)** cells after actinomycin D treatment were examined by RT-qPCR analysis. (**e-f)** The expression of linear and circular HIPK3 in KYSE140 **(e)** and EC9706 **(f)** cells after RNAse R treatment were examined by RT-qPCR analysis. **(g-h)** The knockdown efficiency of si circHIPK3 were detected by RT-qPCR analysis. **(i)** The expression of circHIPK3 as described in Fig. 1Q was detected by RT-qPCR analysis. **(j-m)** KYSE140 cells were transfected with negative control sh RNA (sh NC) or sh RNA targeting circHIPK3 (sh circHIPK3) followed RT-qPCR analysis **(j)**, cell proliferation assay **(k),** and colony formation assay **(l-m).** All experiments were repeated for three times, and representative data is shown (mean ± SD, * P < 0.05, ** P < 0.01, *** P < 0.001).

**Figure S2. The ceRNA network constituting of circHIPK3-miRNAs-mRNAs**

**(a)** The ceRNA network constituting of circHIPK3-miRNAs-mRNAs (genes positively regulated by circHIPK3, n = 51) is shown. Nodes in green, yellow, and light blue represent circHIPK3, miRNAs, and mRNAs, respectively. (**b-c)** The correlation between the expression of circHIPK3 and SCD (**b**) or ACACA (**c**) in ESCC tumor samples (in-house) was analyzed (n = 50).

**Figure S3.** **The interference efficiency of siRNA targeting FASN**

**(a-b)** KYSE140 and EC9706 cells as described in Fig. 3 were subjected to RT-qPCR analysis to examine the expression of FASN.

**Figure S4. Knockdown of miR-637 reverses the effect of circHIPK silencing on FASN expression**

**(a-b)** KYSE140 and EC9706 cells as described in Fig. 4 were subjected to RT-qPCR analysis to examine the expression of FASN.

**Figure S5. circHIPK3 serves as a sponge for miR-637**

**(a-d)** The standard curves for copy number analysis **(a, b)** and the copy numbers of circHIPK3 and miR-637 in KYSE140 **(c)** and EC9706 cells **(d)** were measured by RT-qPCR analysis. **(e-f)** KYSE140 and EC9706 cells as described in Fig. 5 were subjected to RT-qPCR analysis to examine the expression of FASN.

**Figure S6. The interference efficiency of Anti-sense oligonucleotide targeting circHIPK3**

**(a-b)** The knockdown efficiency of ASO-circHIPK3 were detected by RT-qPCR as shown.
