# Supplementary material for: CircHIPK3 regulates fatty acid metabolism through miR-637/FASN axis to promote esophageal squamous cell carcinoma
Source: Cell Death Discov. 2024 Mar 2;10:110. doi: 10.1038/s41420-024-01881-z (PMC10908791; doi:10.1038/s41420-024-01881-z)
Supplement: Supplementary file 2 — Supplementary table legends [file 41420_2024_1881_MOESM2_ESM.docx]

**Supplementary table legends**

**Table S1.** The sequence information for all RT-qPCR primers, shRNAs, siRNAs, miRNA inhibitors, miRNA mimics, and ASOs used in this study.

**Table S2.** The expression of genes positively-regulated by circHIPK3 and related to fatty acid metabolism as described in both Fig. 2i and Fig. S2a (n = 9) in ESCC compared to normal tissue samples in TCGA database.
