# Supplementary material for: CircHIPK3 regulates fatty acid metabolism through miR-637/FASN axis to promote esophageal squamous cell carcinoma
Source: Cell Death Discov. 2024 Mar 2;10:110. doi: 10.1038/s41420-024-01881-z (PMC10908791; doi:10.1038/s41420-024-01881-z)
Supplement: Supplementary file 3 — Table S1 [file 41420_2024_1881_MOESM3_ESM.docx]

| **Table S1.** The sequence information for all RT-qPCR primers, shRNAs, siRNAs, miRNA inhibitors, miRNA mimics, and ASOs used in this study. | | | |  |  |
| --- | --- | --- | --- | --- | --- |
| **RT-qPCR primers** | | |  |  |  |
| circHIPK3 | Forward primer | TCGGCCAGTCATGTATCAAA |  |  |  |
|  | Reverse primer | CCCTTAGTGGGAGGATGAGA |  |  |  |
| GAPDH | Forward primer | CAATGACCCCTTCATTGACC |  |  |  |
|  | Reverse primer | TTGATTTTGGAGGGATCTCG |  |  |  |
| MALAT1 | Forward primer | TGCTGTGTGCCAATGTTTCG |  |  |  |
|  | Reverse primer | AATCCCCTAGGGAAGGGGTC |  |  |  |
| FASN | Forward primer | CCCCTGATGAAGAAGGATCA |  |  |  |
|  | Reverse primer | ACTCCACAGGTGGGAACAAG |  |  |  |
| SCD | Forward primer | CCCAGCTGTCAAAGAGAAGG |  |  |  |
|  | Reverse primer | GGGGGCTAATGTTCTTGTCA |  |  |  |
| ACACA | Forward primer | TGACCTCACTGCCATTCCAT |  |  |  |
|  | Reverse primer | TAACCGTGGGCACAAAGTTG |  |  |  |
| U6 | Forward primer | CGCTTCGGCAGCACATATAC |  |  |  |
|  | Reverse primer | AAAATATGGAACGCTTCACGA |  |  |  |
| ACTIN | Forward primer | CTCTTCCAGCCTTCCTTCCT |  |  |  |
|  | Reverse primer | AGCACTGTGTTGGCGTACAG |  |  |  |
|  |  |  |  |  |  |
| **shRNA targeting sequence** | | |  |  |  |
| sh circHIPK3 | Forward primer | 5’-CCGGGGTACTACAGGTATGGCCTCACTCGAGTGAGGCCATACCTGTAGTACCTTTTTG-3’ |  |  |  |
|  | Reverse primer | 5’-AATTCAAAAAGGTACTACAGGTATGGCCTCACTCGAGTGAGGCCATACCTGTAGTACC-3’ |  |  |  |
|  |  |  |  |  |  |
| **siRNA targeting sequence** | | |  |  |  |
| si circHIPK3 #1 | GGTACTACAGGTATGGCCT | |  |  |  |
| si circHIPK3 #2 | GGTACTACAGGTATGGCCTC | |  |  |  |
| si FASN #1 | GCATCAATGTCCTGCTGAA | |  |  |  |
| si FASN #2 | GCGTTGACCTGGTCTTGAA | |  |  |  |
|  |  |  |  |  |  |
| **ASO targeting sequence** | | |  |  |  |
| ASO circHIPK3 #1 | GGTACTACAGGTATGGCCTC | |  |  |  |
| ASO circHIPK3 #2 | GTACTACAGGTATGGCCTCA | |  |  |  |
|  |  |  |  |  |  |
| **FISH probe targeting sequence** | | |  |  |  |
| FISH-circHIPK3 | 5'-GACTTGTGAGGCCATACCTGTAGTACCGAGATTG-3' | |  |  |  |

| **miRNA sequence** | | |  |  |  |
| --- | --- | --- | --- | --- | --- |
| miR-637 mimic | 5’-ACUGGGGGCUUUCGGGCUCUGCGU-3’  3’-UGACCCCCGAAAGCCCGAGACGCA-5’ | |  |  |  |
| miR-637 inhibitor | 5’-ACGCAGAGCCCGAAAGCCCCCAGU-3’ | |  |  |  |
| miRNA mimic NC | 5’-UUUGUACUACACAAAAGUACUG-3’  3’-AAACAUGAUGUGUUUUCAUGAC-5’ | |  |  |  |
| miRNA inhibitor NC | 5’-CAGUACUUUUGUGUAGUACAAA-3’ | |  |  |  |
|  |  |  |  |  |  |
