# Supplementary material for: CircHIPK3 regulates fatty acid metabolism through miR-637/FASN axis to promote esophageal squamous cell carcinoma
Source: Cell Death Discov. 2024 Mar 2;10:110. doi: 10.1038/s41420-024-01881-z (PMC10908791; doi:10.1038/s41420-024-01881-z)
Supplement: Supplementary file 4 — Table S2 [file 41420_2024_1881_MOESM4_ESM.docx]

| **Table S2.** The expression of genes positively-regulated by circHIPK3 and related to fatty acid metabolism as described in both Fig. 2i and Fig. S2a (n = 9) in ESCC compared to normal tissue samples in TCGA database. | | |
| --- | --- | --- |
| Gene name | Up- or Down-regulated in ESCC | Significant (P < 0.05), Yes or No |
| **FASN** | **Up** | **Yes** |
| KIF5B | Up | No |
| RAPGEF5 | Up | Yes |
| PEX19 | Up | No |
| ACACA | Up | Yes |
| SLC7A5 | Up | No |
| SCD | Up | Yes |
| GPD1L | Down | Yes |
| MAPK9 | Up | No |
