# Supplementary figures and images for: CircHIPK3 regulates fatty acid metabolism through miR-637/FASN axis to promote esophageal squamous cell carcinoma
Source: Cell Death Discov. 2024 Mar 2;10:110. doi: 10.1038/s41420-024-01881-z (PMC10908791; doi:10.1038/s41420-024-01881-z)

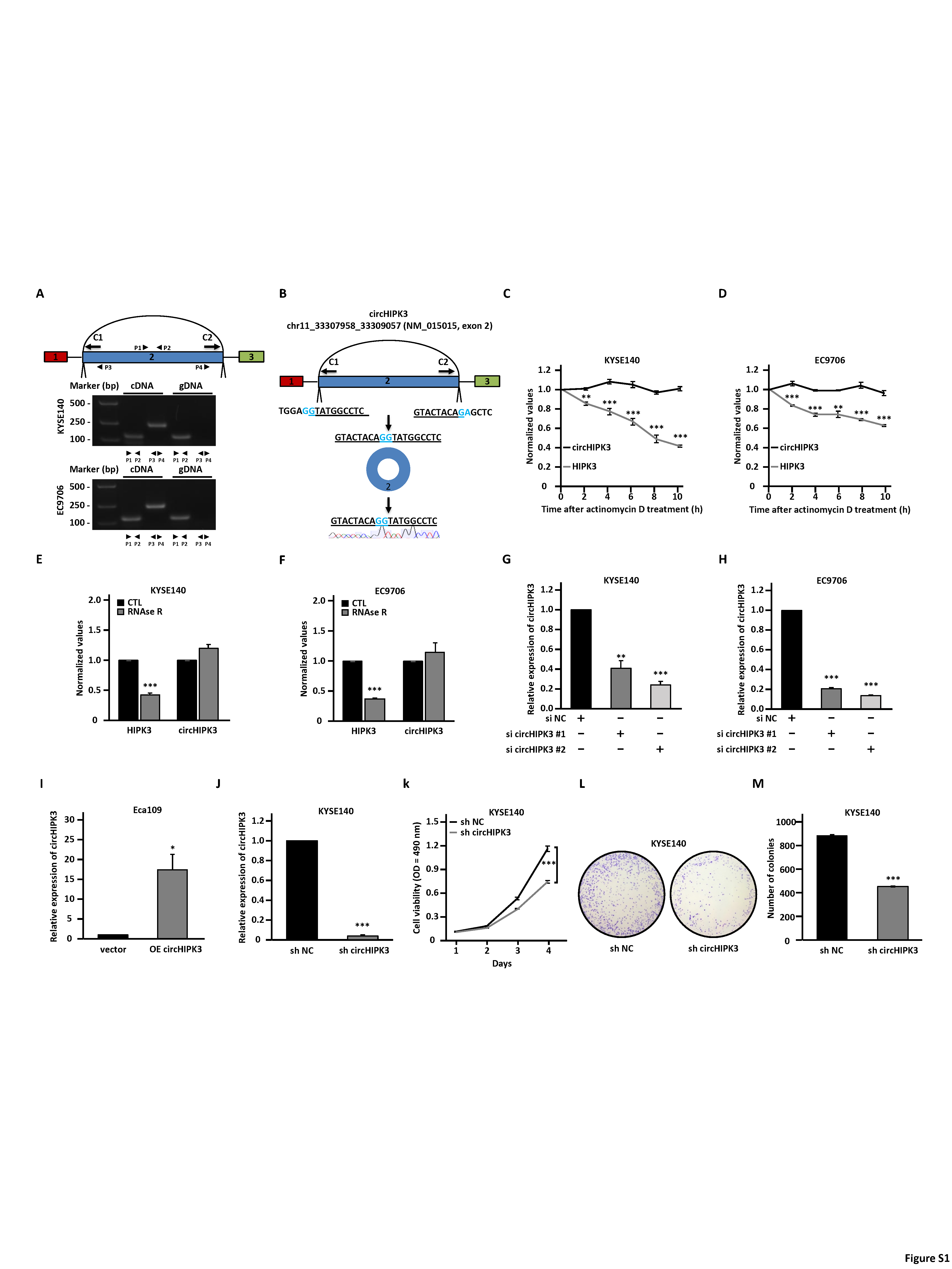

Supplement: Supplementary file 5 — Fig. S1 [file 41420_2024_1881_MOESM5_ESM.png]

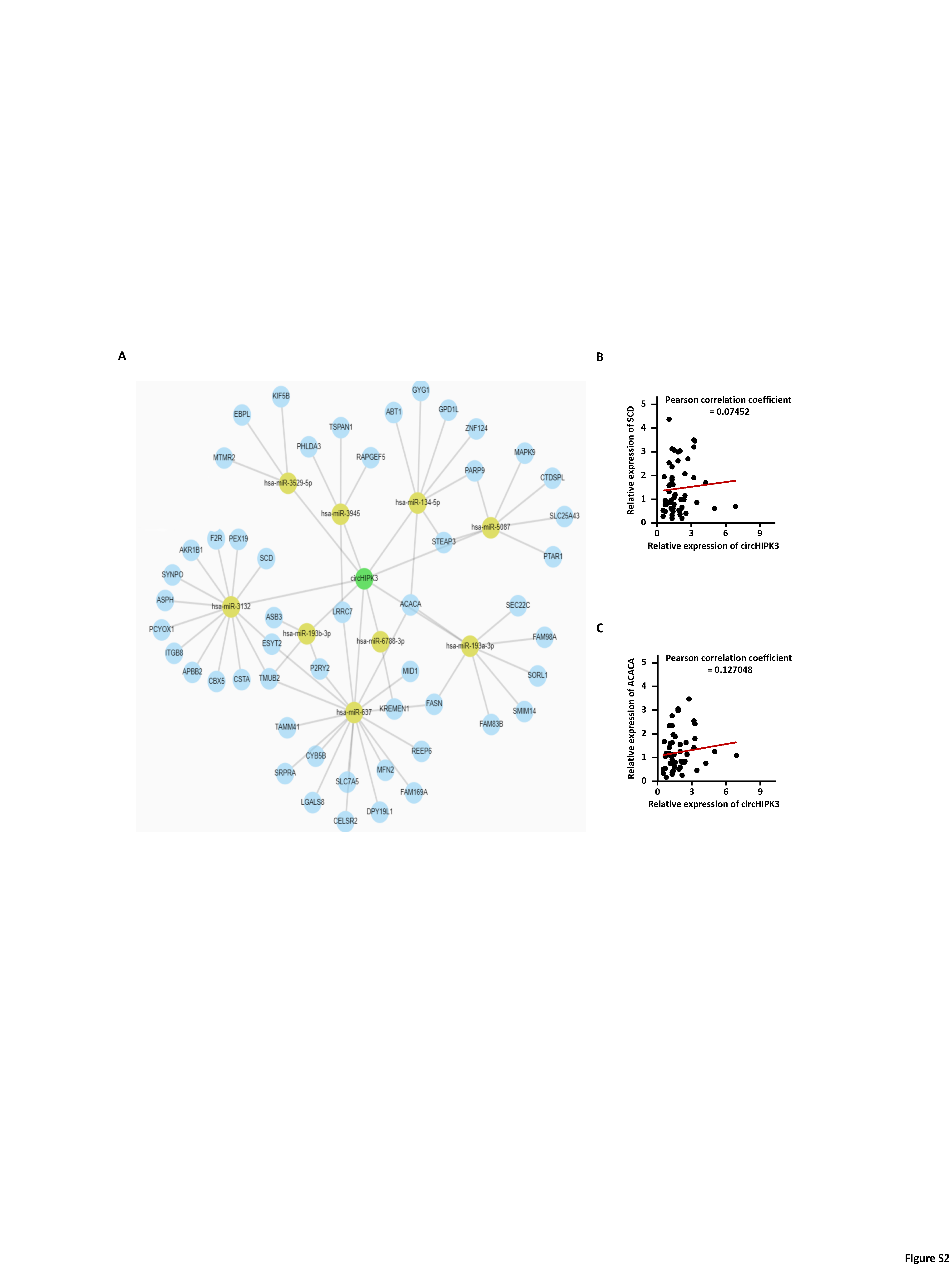

Supplement: Supplementary file 6 — Fig. S2 [file 41420_2024_1881_MOESM6_ESM.png]

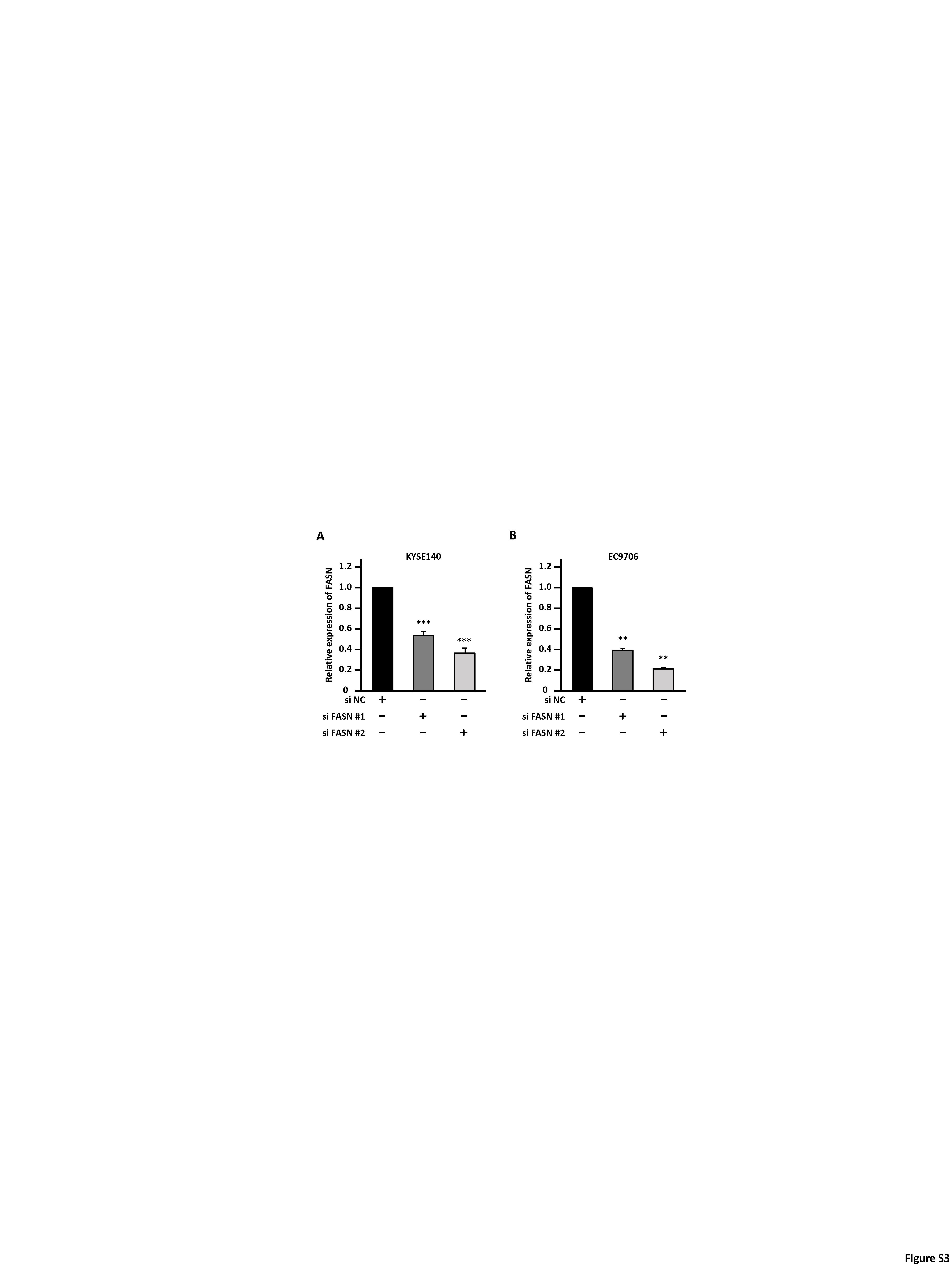

Supplement: Supplementary file 7 — Fig. S3 [file 41420_2024_1881_MOESM7_ESM.png]

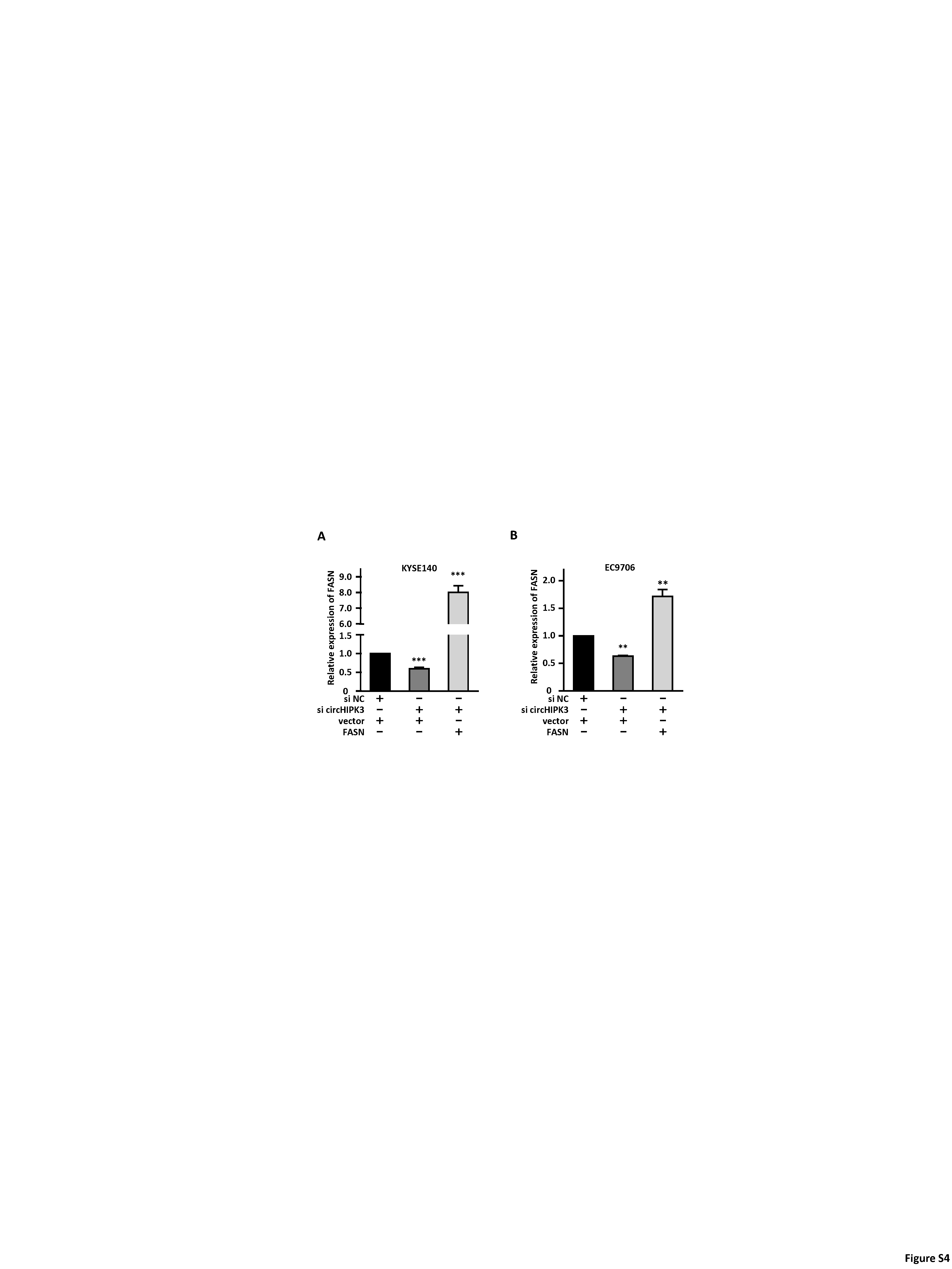

Supplement: Supplementary file 8 — Fig. S4 [file 41420_2024_1881_MOESM8_ESM.png]

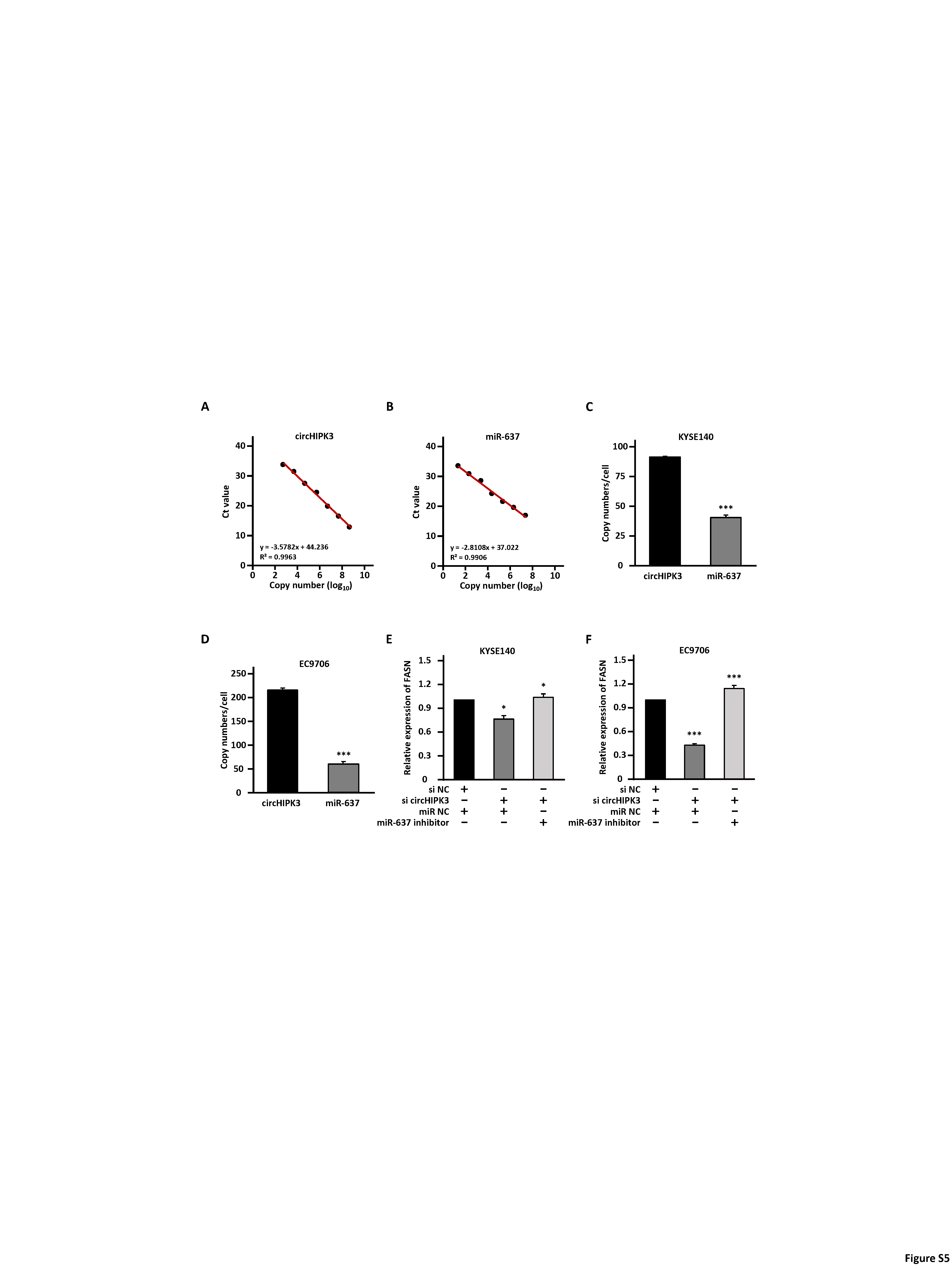

Supplement: Supplementary file 9 — Fig. S5 [file 41420_2024_1881_MOESM9_ESM.png]

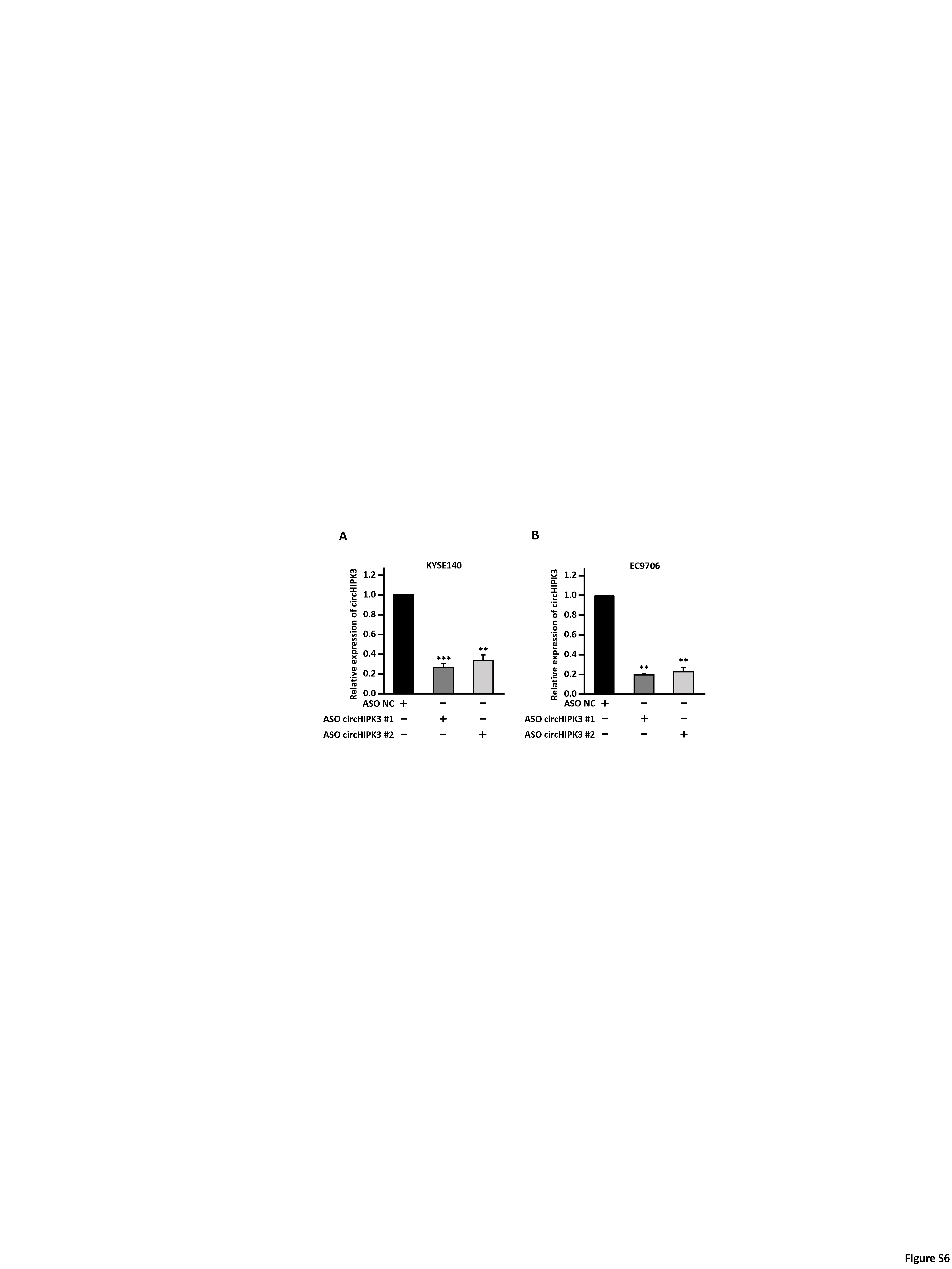

Supplement: Supplementary file 10 — Fig. S6 [file 41420_2024_1881_MOESM10_ESM.png]
