# Supplementary material for: CircHIPK3 regulates fatty acid metabolism through miR-637/FASN axis to promote esophageal squamous cell carcinoma
Source: Cell Death Discov. 2024 Mar 2;10:110. doi: 10.1038/s41420-024-01881-z (PMC10908791; doi:10.1038/s41420-024-01881-z)

Figure 2M

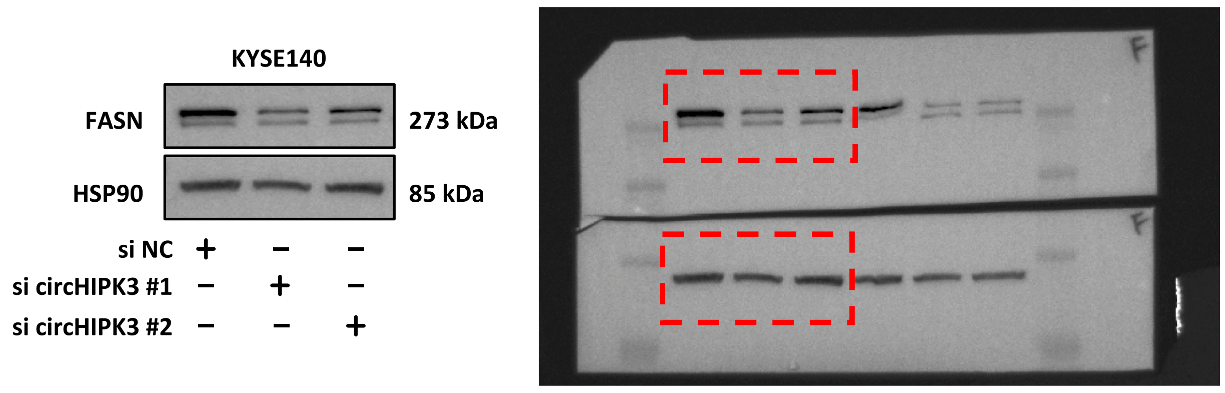

Figure 2N

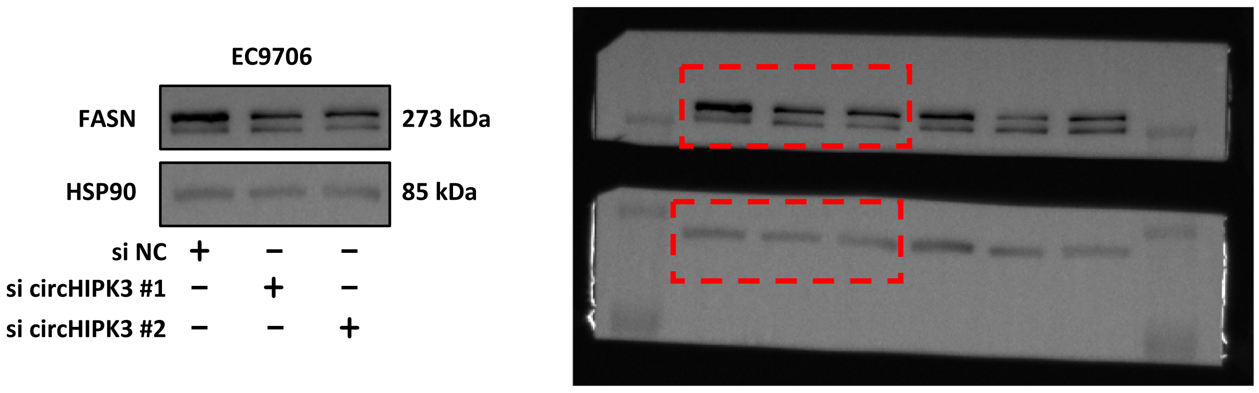

Figure 3A

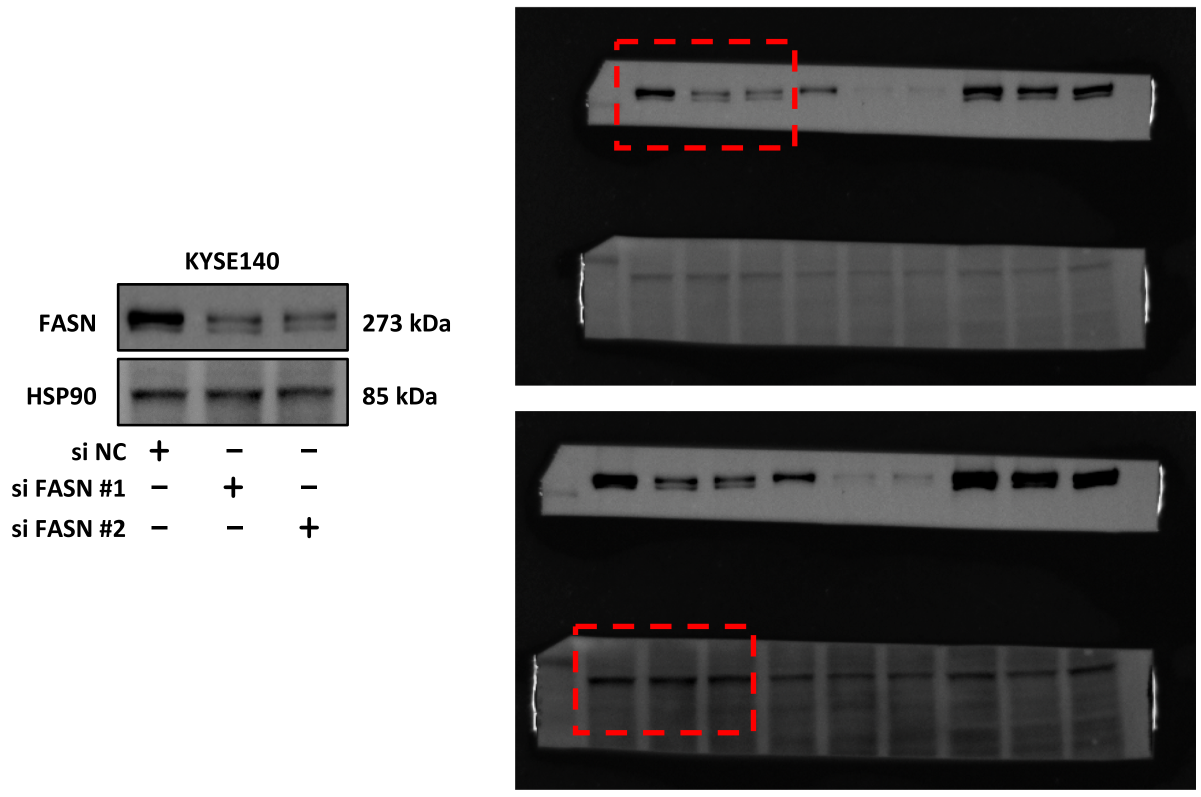

Different exposure depths of the same film.

Figure 3B

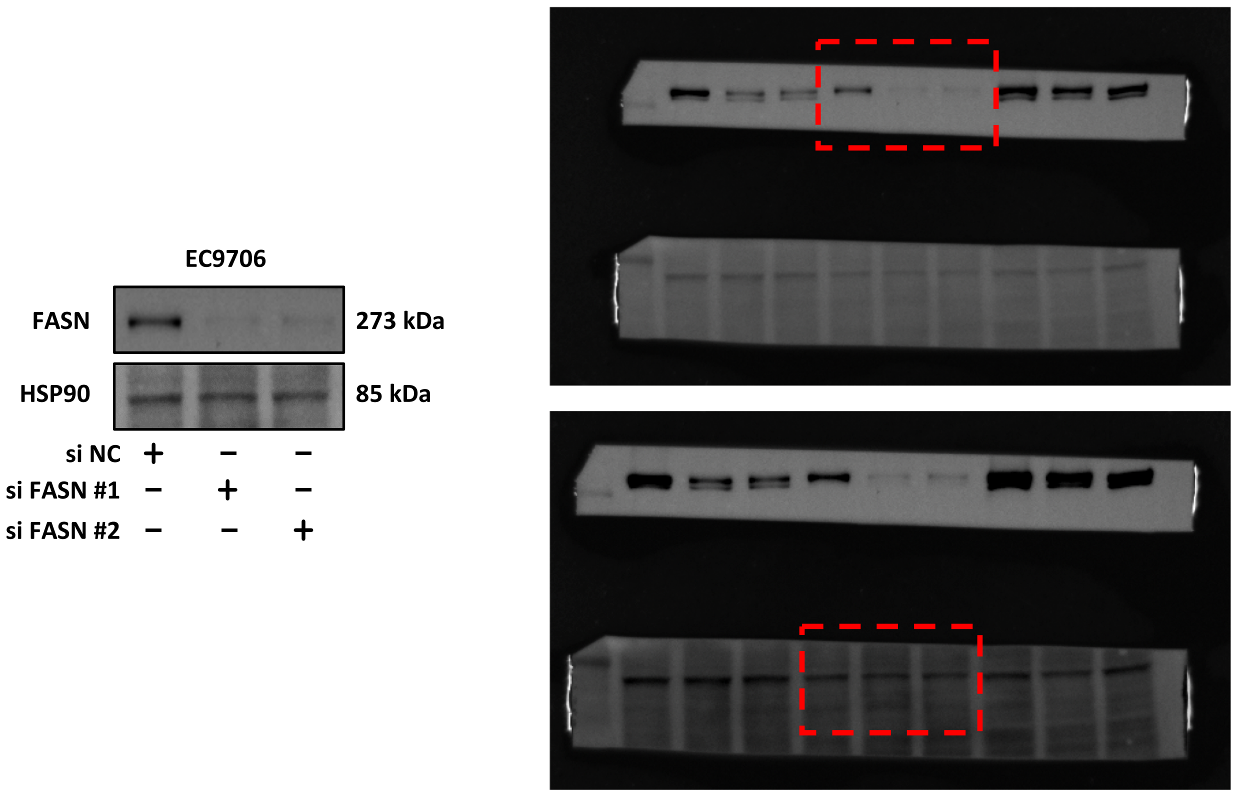

Different exposure depths of the same film.

Figure 4A

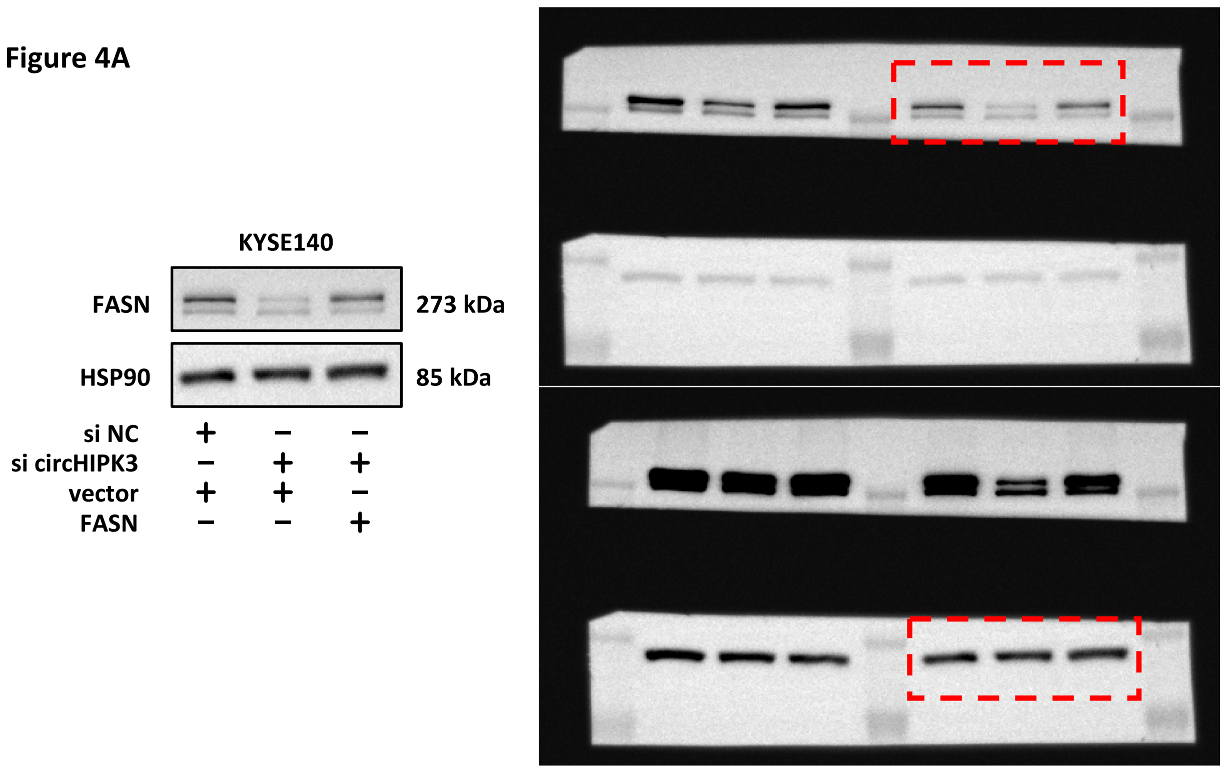

Different exposure depths of the same film.

Figure 4B

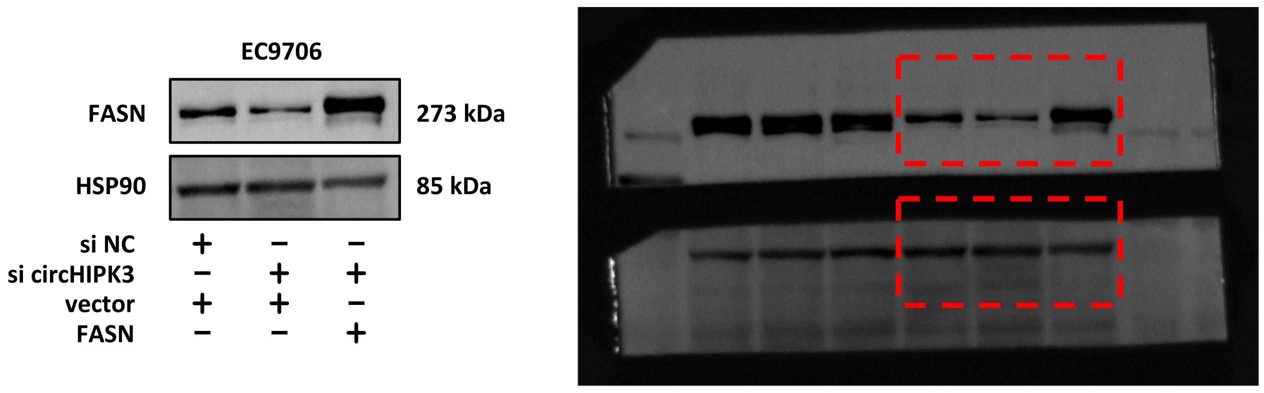

Figure 5F

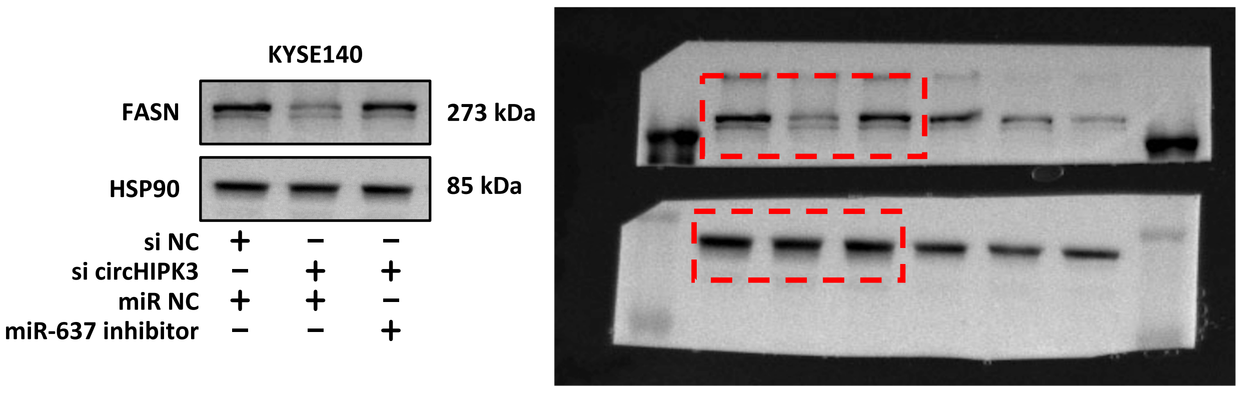

Figure 5G

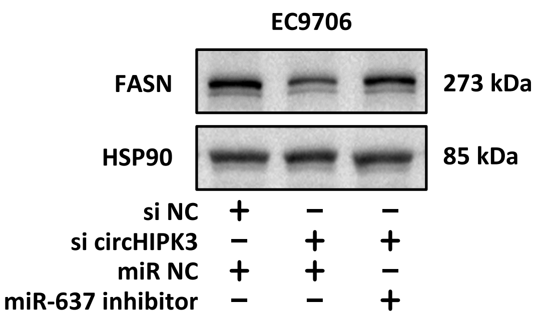

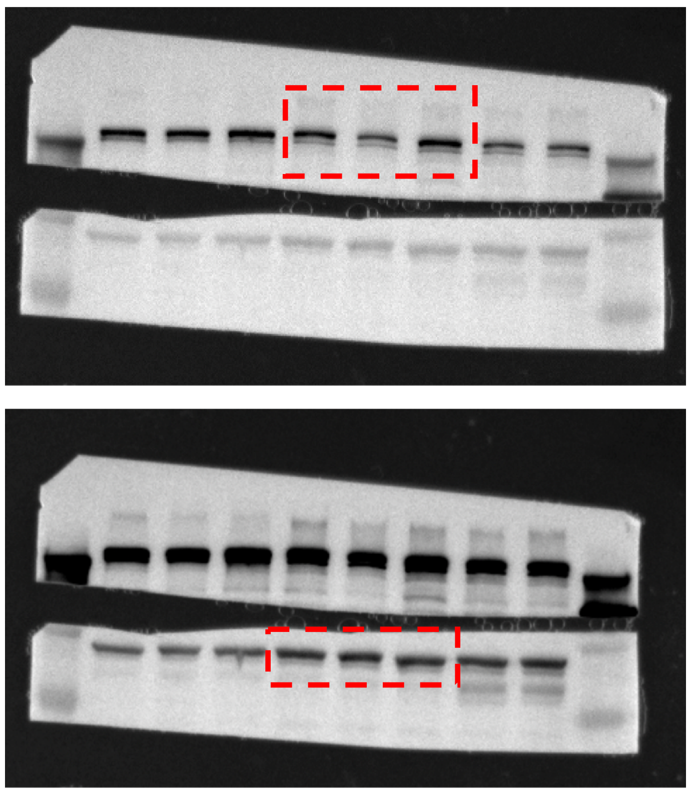

Different exposure depths of the same film.

Figure 6A

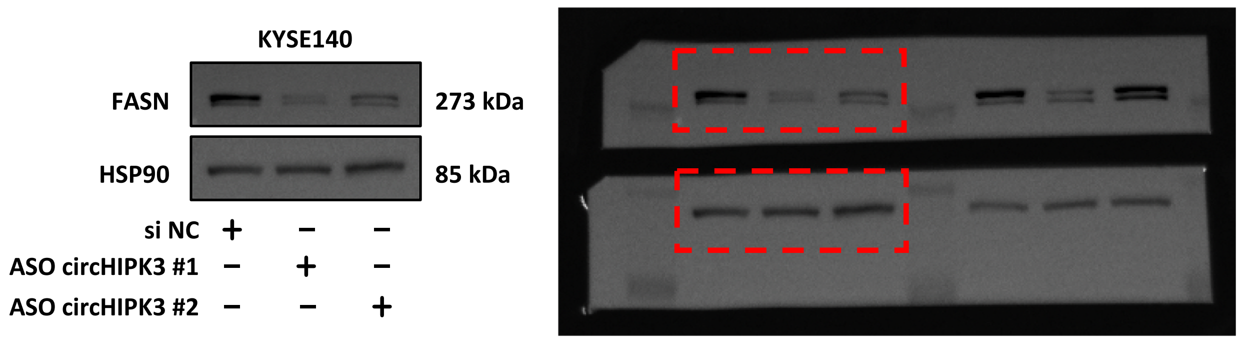

Figure 6B

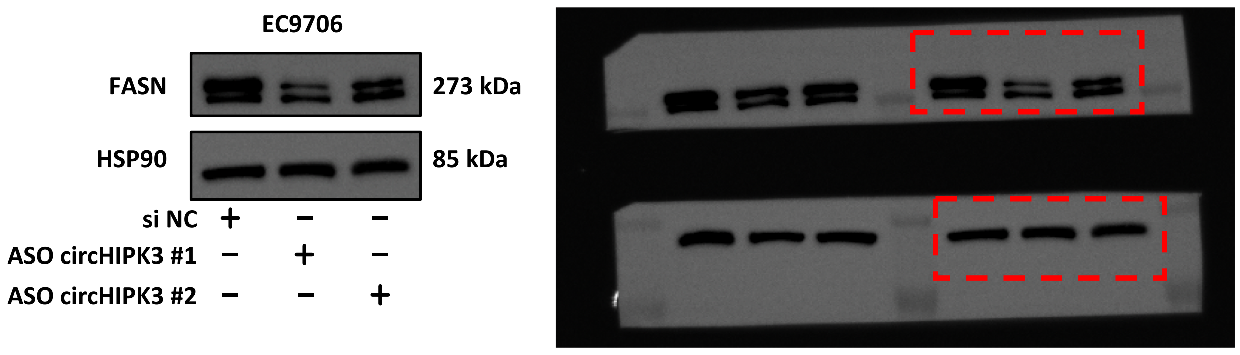

Supplement: Supplementary file 11 — Original Data File [file 41420_2024_1881_MOESM11_ESM.pdf]
